# Near-infrared fluorescence lifetime imaging: fluorophores, methods, and applications

**DOI:** 10.1117/1.JBO.31.10.100901

**Published:** 2026-10-21

**Authors:** Nanxue Yuan, Catherine Sherry, Navid Ibtehaj Nizam, Saif Ragab, Luis Chavez, Vikas Pandey, Amit Verma, Colleen N. Scott, Margarida M. Barroso, Xavier Intes

**Affiliations:** a Rensselaer Polytechnic Institute, Center for Modeling, Simulation and Imaging in Medicine, Troy, New York, United States; b Albany Medical College, Department of Molecular and Cellular Physiology, Albany, New York, United States; c West Chester University, Department of Biomedical Engineering, West Chester, Pennsylvania, United States; d Mississippi State University, Department of Chemistry, Starkville, Mississippi, United States

**Keywords:** fluorescence lifetime imaging, near-infrared fluorescence, near-infrared fluorophores, multiplexed imaging, Förster resonance energy transfer, microenvironmental sensing, fluorescence-guided surgery, indocyanine green, clinical translation

## Abstract

**Significance:**

Near-infrared (NIR) fluorescence lifetime imaging (FLI) provides molecular and functional contrast that encodes local biochemical and biophysical environments beyond what fluorescence intensity alone can reveal. Realizing this potential across biological imaging and emerging clinical applications requires rigorous fluorophore characterization and a critical assessment of the photophysics, measurement capabilities, and conditions governing reliable lifetime interpretation.

**Aim:**

We comprehensively survey the contemporary NIR FLI landscape, spanning fluorophore photophysics, imaging instrumentation, lifetime analysis, and biomedical applications, to identify application-specific criteria for fluorophore selection, quantitative measurement, and translational validation.

**Approach:**

Within the NIR range, we critically compare organic dyes, fluorescent proteins, inorganic fluorophores, and endogenous emitters, integrating photophysical evidence with acquisition and analysis methods for multiplexing, Förster resonance energy transfer (FRET), environmental sensing, and clinical imaging.

**Results:**

Reported NIR fluorophore lifetimes span tens of picoseconds to over 100 ns and depend on molecular structure, solvent, binding state, and aggregation. Multiplexing requires reproducible, distinguishable decay signatures; sensing requires selective, calibrated lifetime responses; and FRET requires reliable donor-only references. Uncorrected instrumental effects, tissue photon migration, and inappropriate analysis assumptions can distort lifetime estimates. Incomplete characterization and inconsistent reporting further limit reproducibility and comparison across studies.

**Conclusions:**

Quantitative NIR FLI requires precise probe characterization, standardized measurement validation, and application-specific performance assessment. We provide practical recommendations for probe selection, lifetime contrast interpretation, and clinical utility evaluation, establishing a framework for reproducible NIR FLI from mechanistic cell studies to image-guided interventions.

## 1 Introduction

Fluorescence lifetime τ, the average time a fluorophore spends in its excited state before deactivation, reflects the combined radiative and nonradiative decay pathways illustrated in the Jablonski diagram and Förster resonance energy transfer (FRET) energy transfer scheme in Fig. 1.^1^^–^^5^ In contrast to fluorescence intensity, which is strongly influenced by probe concentration, excitation power, and tissue optical properties, fluorescence lifetime is an intrinsic property of a fluorophore within a defined microenvironment. Depending on the fluorophore and its underlying photophysical mechanism, fluorescence lifetime can be modulated by solvent polarity, binding state, aggregation, pH, quenching, temperature, or oxygenation.^4^^,^^6^^–^^8^ These dependencies allow fluorescence lifetime to report local molecular environments rather than serving merely as a source of signal variability. Fluorescence lifetime imaging (FLI) maps fluorescence decay across space, providing contrast associated with fluorophore identity, molecular state, and the surrounding biochemical or biophysical environment.

**Fig. 1:**
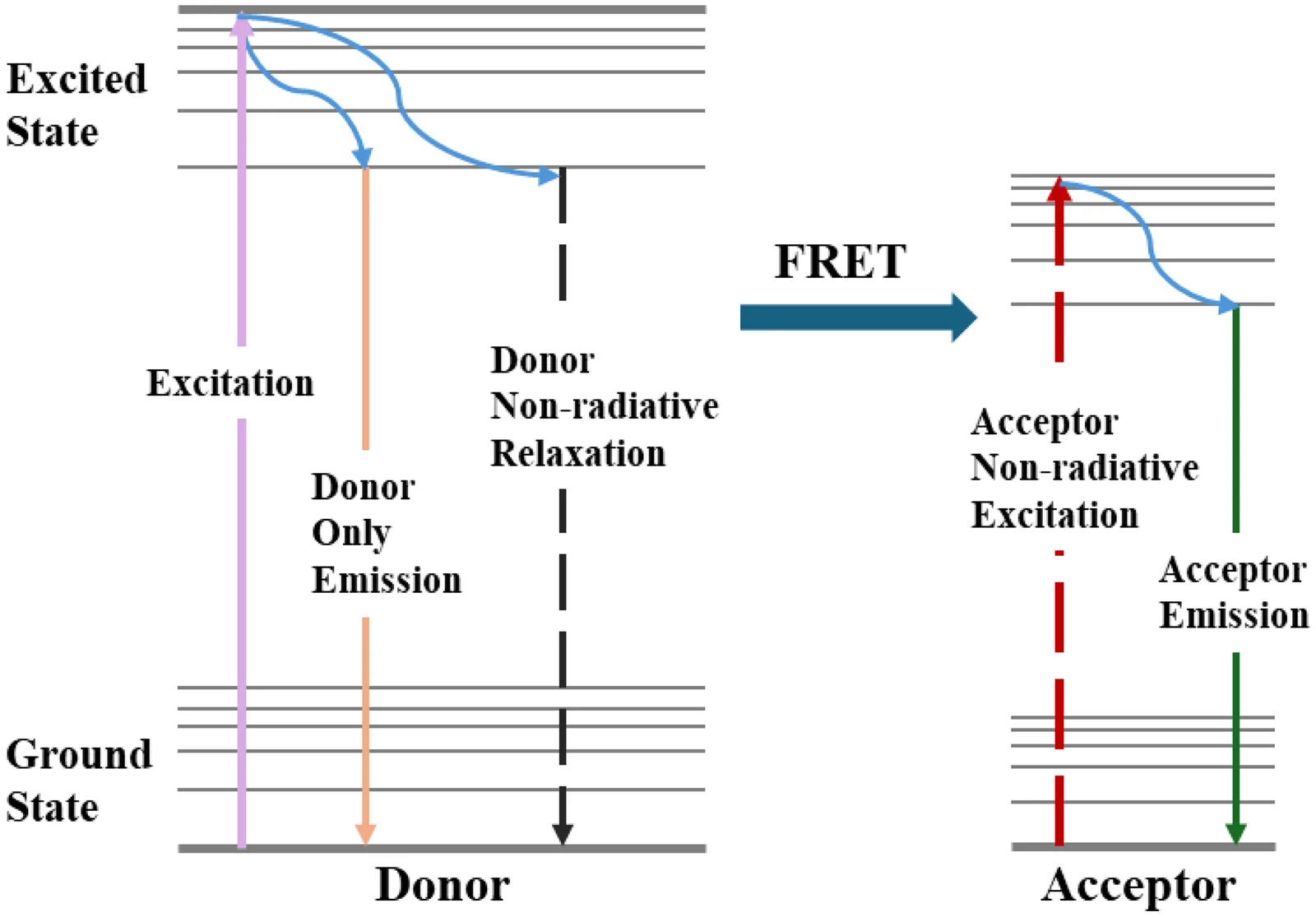
Schematic illustration of the excited-state pathways underlying fluorescence emission and Förster resonance energy transfer (FRET).

The near-infrared (NIR) region (650 to 950 nm) provides a biological optical window defined by the wavelength-dependent contributions of tissue absorption, scattering, autofluorescence (AF), fluorophore brightness, and detector sensitivity.^9^^–^^11^ Within this range, hemoglobin absorption and tissue autofluorescence are generally reduced relative to much of the visible spectrum while scattering decreases with increasing wavelengths.^9^^,^^12^ However, beyond this conventional NIR window, water absorption and limitations in fluorophore brightness and detector sensitivity become increasingly important.^10^^,^^11^^,^^13^ This wavelength-dependent balance improves photon penetration and supports fluorescence imaging across intact organs and small animals.^11^^,^^13^

Combining lifetime-resolved contrast with the optical advantages of NIR wavelengths expands the reach of FLI across biomedical research. In cells and tissues at the microscopic scale, NIR FLI can resolve fluorophore identity, molecular state, microenvironment, and molecular interactions. At the macroscopic scale, NIR FLI extends these lifetime-resolved measurements from cell-based assays to intact organs and living animals, connecting mechanistic cell and molecular studies with noninvasive preclinical molecular imaging and emerging clinical applications, including molecular diagnostics and image-guided interventions. Realizing this potential requires fluorophores, with lifetime behavior that is rigorously characterized under relevant biological and measurement conditions.

For a fluorophore in a fixed molecular state, changes in probe concentration or excitation power primarily scale the detected photon count without altering the normalized fluorescence decay, provided that aggregation, self-quenching, reabsorption, saturation, photobleaching, and pile-up are negligible.^14^ In tissue, however, depth-dependent photon migration and temporally overlapping background fluorescence can distort the measured decay, whereas detector timing response and temporal sampling limit recovery of short decay components.^15^ These effects are particularly consequential for sub-nanosecond NIR fluorophores and must be distinguished from genuine environment-dependent changes in fluorescence lifetime.

Precise fluorophore characterization is critical to the successful implementation and continued advancement of quantitative NIR FLI. Reported lifetimes can vary substantially across solvent environments, binding and aggregation states and temperatures.^16^^–^^18^ Differences in excitation and detection wavelengths, detector response, acquisition parameters, and lifetime-analysis methods further complicate cross-study interpretation. These factors can obscure whether an observed difference arises from fluorophore behavior, the biological environment, or the measurement process itself. Fluorophore selection must therefore balance photophysical performance, biological compatibility, instrument constraints, and application-specific lifetime behavior and should be supported by transparently annotated reference data. This is particularly important in the context of clinical translation, where repeatability and reproducibility are essential.

Fluorophore design and selection for NIR FLI present distinct challenges. As fluorophore emission is shifted from the visible region toward the NIR, the smaller electronic energy gap promotes nonradiative relaxation, contributing to the relatively low quantum yields and short fluorescence lifetimes exhibited by many NIR fluorophores.^19^^,^^20^ Endogenous fluorophores generally exhibit weak NIR emission and overlapping spectral signatures while their fluorescence lifetimes are strongly modulated by binding state and metabolic context. This sensitivity supports metabolic FLI approaches but can limit molecular specificity and absolute quantification in applications requiring target-specific contrast.^21^^,^^22^ Organic NIR dyes, including cyanine, BODIPY, and xanthene derivatives, exhibit lifetimes highly sensitive to polarity, viscosity, and aggregation.^23^^,^^24^ Engineered fluorescent proteins that exploit biliverdin-binding chromophores, such as miRFPs and iRFPs, offer genetically encoded NIR fluorescence but often display modest quantum yields and environment-dependent lifetime behavior.^25^ Finally, inorganic emitters, such as quantum dots or lanthanide-doped nanocrystals, provide longer fluorescence lifetimes but raise concerns regarding cytotoxicity and biocompatibility.^26^

NIR FLI extends molecular imaging beyond fluorophore localization by resolving molecular states, interactions, and environments. NIR FLI-FRET reports molecular proximity through donor-lifetime quenching while lifetime-based multiplexing separates probes with overlapping emission spectra but distinct fluorescence decay kinetics.^16^^,^^27^^–^^29^ Microscopic NIR FLI-FRET provides controlled validation of probe behavior and lifetime components, supporting quantitative macroscopic FLI-FRET measurements in intact animals. Together with microenvironmental sensing, these approaches connect basic mechanistic studies with preclinical molecular imaging. Although NIR FLI has been demonstrated in multiplexing, FRET, environmental sensing, preclinical small animal imaging, and early clinical tissue characterization, broader clinical translation remains limited. Most fluorescence-guided surgery (FGS) systems still rely primarily on fluorescence intensity rather than lifetime-resolved contrast.^30^^–^^32^ Accordingly, this review prioritizes applications in which fluorescence lifetime was directly measured and discusses intensity-only studies when they provide relevant clinical or translational context.

Focusing on fluorophores relevant to quantitative NIR FLI across cells, tissues, and living subjects, this review critically evaluates the photophysical properties and reported lifetimes of exogenous organic dyes, fluorescent proteins, inorganic emitters, and endogenous fluorophores. Particular emphasis is placed on how measurement conditions, instrumentation, and analytical methods influence lifetime values and their interpretation in lifetime-resolved applications. The review examines NIR photophysics and fluorophore classes, followed by applications in multiplexing, FRET-based molecular interaction imaging, microenvironmental sensing, and clinical translation. Subsequent sections evaluate acquisition modalities, detector architectures, computational analysis methods, barriers to reproducible quantification, and pathways toward standardization. By integrating fluorophore design, photophysical properties, and reported lifetime characteristics with imaging instrumentation, analysis, and application-specific evidence, this review defines key requirements for the successful implementation and continued advancement of quantitative NIR FLI.

The fluorescence lifetime values summarized in Tables 1–5 were compiled from reports obtained under different solvent environments, binding states, temperatures, and instrumentation and analytical methods. Peer-reviewed sources were prioritized, and manufacturer datasheets or preprints included only when they directly supported a reported photophysical or lifetime value. These values should therefore be interpreted as representative reported ranges rather than directly comparable measurements, further emphasizing the need for standardized reporting in NIR fluorescence lifetime studies.

## 2 NIR-Specific Photophysics

NIR fluorophores present unique photophysical challenges. In particular, photons with longer wavelengths necessarily possess lower energy, as described by Planck’s relation:

$$E=\frac{hc}{\lambda },$$ (1)

where E is energy, h is Planck’s constant, c is the speed of light, and λ is wavelength. As the λ increases, the energy gap decreases resulting in molecules fluorescing in the NIR window having a smaller energy gap between the highest occupied molecular orbital (HOMO) and lowest unoccupied molecular orbital (LUMO).^113^^–^^116^

Fluorescence lifetime (τ) is governed by the competition between radiative and nonradiative decay processes and can be expressed as

$$\tau =\frac{1}{k_{r}+k_{\mathrm{nr}}},$$ (2)

where $k_{r}$ and $k_{\mathrm{nr}}$ are the rate constants for radiative decay (photon emission) and nonradiative decay, respectively.^117^ Quantum yield (Φ) is similarly defined as

$$\Phi =\frac{k_{r}}{k_{r}+k_{\mathrm{nr}}}.$$ (3)

For NIR fluorophores, the narrower HOMO–LUMO gap leads to increased coupling between electronic and vibrational states, resulting in elevated $k_{\mathrm{nr}}$.^19^^,^^20^ This provides the fundamental photophysical basis for the reduced quantum yields and short fluorescence lifetimes commonly observed in NIR fluorophores. Several molecular design strategies have therefore been employed to achieve NIR emission while minimizing $k_{\mathrm{nr}}$, including incorporating donor–acceptor architectures,^118^ and rigidifying the fluorophore scaffold.^119^^–^^121^

Interestingly, although elevated $k_{\mathrm{nr}}$ is disadvantageous in terms of brightness, it also renders many NIR fluorophores highly sensitive to their local environment, making them particularly valuable as biological sensors. This is especially relevant in the context of fluorescence lifetime imaging (FLI) because fluorescence lifetime measurements are directly sensitive to changes in $k_{\mathrm{nr}}$ while remaining largely independent of fluorophore concentration and excitation intensity. For example, the fluorescence lifetime of some NIR fluorophores is sensitive to solvent polarity, as polar solvents can reorient around the excited-state fluorophore, stabilize charge-separated configurations, and increase $k_{\mathrm{nr}}$ through solvent relaxation.^17^ Similarly, fluorophore interactions with local proteins, changes in viscosity, and alterations in pH can significantly influence excited-state stability. These interactions between the dye and its environment often restrict intramolecular motion, suppress nonradiative pathways, and thereby increase fluorescence lifetime.^122^^,^^123^ Aggregation can lead to either fluorescence quenching (aggregation-caused quenching, ACQ) or fluorescence enhancement (aggregation-induced emission, AIE), depending on molecular structure, leading to a broad range of sensing and imaging applications.^124^^,^^125^ Finally, pH-dependent protonation of dyes can alter their electronic structures and excited-state stability, thus enabling the development of pH-sensitive biosensors.^126^

Recent fluorophore development increasingly relies on rational structural modification of established chromophore scaffolds to tune photophysical and biological properties. By engineering the conjugated backbone and terminal groups, key parameters such as emission wavelength, fluorescence lifetime, and quantum yield can be systematically controlled. Longer fluorescence lifetimes in the NIR region have been achieved through structural modification of chromophore scaffolds, particularly rigidification and planarization, which restrict intramolecular rotation and torsional relaxation, suppress internal conversion, and thereby reduce $k_{\mathrm{nr}}$.^77^^,^^127^^,^^128^

Probe suitability depends on the intended role of lifetime contrast. For probe identification and multiplexing, the priority is reproducible separation between fluorescence decay signatures under the anticipated labeling and biological conditions. For environmental sensing, the priority is a reproducible response to the variable of interest, with limited interference from other variables. Environmental sensitivity can therefore support sensing while complicating probe identification. The following sections assess fluorophores in relation to these distinct requirements.

## 3 Major NIR Fluorophore Classes and Their Lifetime Properties

### 3.1 Exogenous Fluorophores: Organic Fluorophores

Organic small-molecule fluorophores form a major foundation of NIR FLI because of their tunable molecular structures, relatively high quantum yields, well-characterized photophysical behavior, and compatibility with bioconjugation or pharmaceutical modification.^77^^,^^129^^–^^132^ Their structures can be engineered to adjust excitation and emission wavelengths, brightness, solubility, targeting properties, and fluorescence lifetime. As a result, organic NIR fluorophores represent one of the most mature and widely used classes of emitters for molecular interaction imaging, target-engagement studies, microenvironmental sensing, and preclinical or clinical fluorescence-guided imaging.

However, many conventional NIR dyes exhibit relatively short lifetimes (typically <1  ns), susceptibility to aggregation-induced quenching, and environmental sensitivity that complicates quantitative lifetime measurements in complex biological environments.^77^^,^^133^^,^^134^ These limitations have motivated the development of structurally rigid, chemically stable, and functionally tunable dye platforms capable of supporting longer and more environment-responsive lifetimes.

The fluorescence lifetime behavior of organic fluorophores is closely linked to the chromophore scaffold, which governs molecular rigidity, conjugation, and nonradiative relaxation pathways. Representative organic NIR chromophore frameworks include polymethine cyanines, such as Cy5–Cy7 and IR-1061,^135^^–^^143^ aza-BODIPYs,^144^^,^^145^ BBTD-based structures,^146^^,^^147^ phthalocyanine,^148^^–^^150^ and xanthene derivatives,^151^ as illustrated in Fig. 2.

**Fig. 2:**
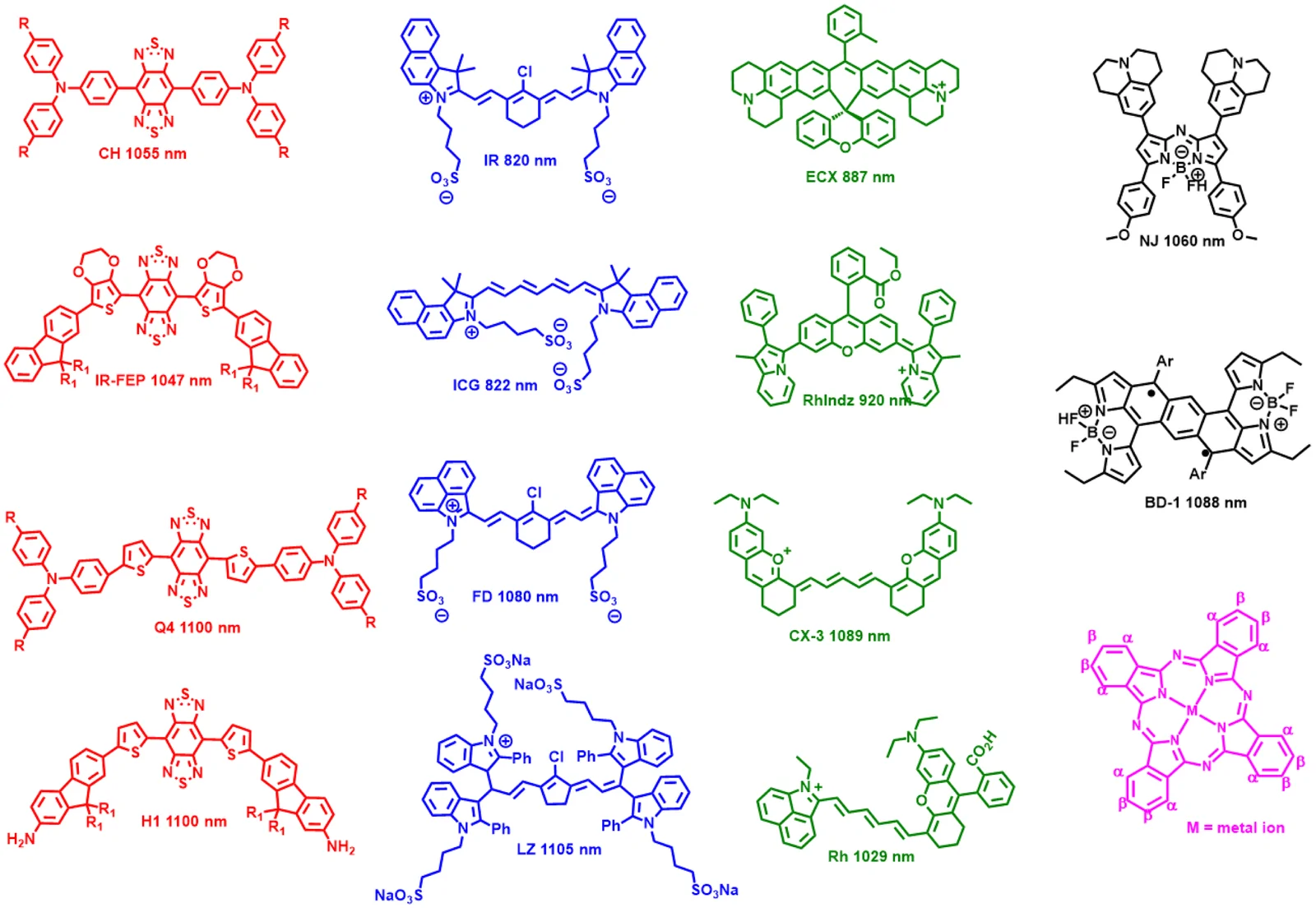
Representative structures of NIR dyes with different chromophore structures. BBTD (red), cyanine (blue), xanthene (green), aza-BODIPY (black), and metallophthalocyanine (fuchsia).

Cyanine dyes represent the most widely used class of exogenous NIR fluorophores and consist of two nitrogen-containing heterocycles linked by a polymethine bridge, forming a highly conjugated π-electron system^152^^–^^154^ shown in Fig. 2. Increasing polymethine chain length not only enhances π-delocalization and redshifts absorption into the NIR region but also increases structural flexibility, promoting nonradiative decay through intramolecular rotation and vibration.^155^^,^^156^ As a result, cyanine dyes typically exhibit short fluorescence lifetimes in the sub-nanosecond to ∼1  ns range due to efficient internal conversion and photoisomerization along the polymethine chain. Charge delocalization along the polymethine chain reduces bond-length alternation and facilitates rapid excited-state deactivation, whereas photoisomerization further contributes to energy dissipation and shortened lifetimes.^157^ Common cyanine derivatives such as Cy5, Cy5.5, and Cy7 remain archetypal NIR emitters with strong molar absorptivity ($\varepsilon \approx 2–3\times 10^{5}\;M^{-1}\;{\mathrm{cm}}^{-1}$). Structural optimization has followed complementary approaches, sulfonation improves aqueous solubility and limits aggregation, whereas conformational constraint, rigidified polymethine bridges, and bis-cyanine architectures restrict intramolecular motion and photoisomerization without further extending conjugation length. Together, these modifications can improve photostability, quantum yield, and fluorescence lifetime characteristics, supporting multiplexed FLI within overlapping spectral regions.^54^^,^^55^^,^^59^^,^^61^^,^^158^^–^^160^ Fluorescence lifetime in cyanine dyes also depends strongly on their microenvironment. For example, protein binding, as observed for ICG, or increased microenvironmental viscosity can restrict polymethine rotation and prolong fluorescence lifetime, whereas aggregation in aqueous environments typically enhances nonradiative quenching and reduces fluorescence lifetime.^161^^,^^162^

Phthalocyanines are highly conjugated macrocyclic fluorophores composed of four isoindole units linked through nitrogen atoms, forming a planar pi-delocalized system^148^^–^^150^ shown in Fig. 2. This structural rigidity suppresses vibrational relaxation and supports relatively stable excited states compared with flexible fluorophores. Their photophysical properties are strongly dependent on the coordinated central metal ion.^163^ In particular, metallophthalocyanines containing diamagnetic metals such as ${\mathrm{Zn}}^{2+}$, ${\mathrm{Al}}^{3+}$, and ${\mathrm{Ga}}^{3+}$ exhibit high triplet quantum yields and long triplet lifetimes, whereas paramagnetic metals such as ${\mathrm{Cu}}^{2+}$ and ${\mathrm{Fe}}^{2+}$ result in reduced excited-state lifetimes and lower photochemical activity.^163^^,^^164^ In addition, phthalocyanines tend to form aggregates in aqueous environments due to strong intermolecular π–π interactions, which decreases fluorescence quantum yield and affects their photophysical behavior.^164^ Chemical modifications, including sulfonation and the use of nanocarriers, are therefore commonly employed to improve solubility and reduce aggregation effects.^165^

Rhodamine dyes are derived from the xanthene scaffold, a tricyclic π-conjugated structure composed of two aromatic rings bridged by a central heteroatom (typically oxygen) and functionalized with electron-donating dialkylamino groups that enable strong fluorescence.^166^^–^^168^ Examples of rhodamine analogs are shown in Fig. 2. However, most classical rhodamine derivatives absorb and emit in the visible spectral range and are therefore not intrinsically NIR fluorophores.^169^ NIR emission is achieved through structural modification of the xanthene core, particularly heteroatom substitution (e.g., Si replacing O), π-extension via benzannulation and donor-acceptor-donor approach,^151^^,^^170^ which extend conjugation and increase molecular rigidity.^167^^,^^171^ Increased rigidity suppresses intramolecular nonradiative relaxation, supporting more stable excited states and mitigating the enhanced nonradiative decay expected at longer wavelengths according to the energy gap law. As a result, rhodamine-derived NIR fluorophores can maintain relatively stable fluorescence lifetimes compared with flexible polymethine systems.^172^ Fluorescence lifetime is further modulated by the biological environment, for example, Rhodamine 800 exhibits longer lifetimes in plasma and whole blood than in aqueous buffer, consistent with restricted molecular motion in protein-bound or high-viscosity environments, whereas aggregation or unfavorable solvent interactions can shorten lifetime.^173^

BODIPY fluorophores are based on a dipyrromethene core coordinated to a boron center, forming a rigid and highly conjugated chromophore with strong absorption, high fluorescence quantum yield, and good photostability.^174^^,^^175^ However, most classical BODIPY dyes emit in the visible range with small Stokes shifts, limiting their applicability for deep-tissue imaging.^176^^,^^177^ To enable NIR emission, structural modifications such as π-extension, donor–acceptor substitution, rigidification, and replacement of the meso-carbon with nitrogen have been developed. In particular, aza-BODIPY derivatives exhibit bathochromic shifts of ∼90 to 100 nm, enabling absorption and emission in the NIR region.^178^^–^^181^ These structural features also govern fluorescence lifetime behavior, as shown in Fig. 2. The rigid BODIPY scaffold suppresses intramolecular motion and reduces nonradiative decay, supporting relatively long fluorescence lifetimes compared with flexible polymethine systems. Substitution patterns and electronic effects, including push–pull architectures, can further tune the energy gap and excited-state dynamics.^182^^,^^183^ By contrast, incorporation of heavy atoms, e.g., Br or I, enhances intersystem crossing and reduces fluorescence quantum yield by promoting triplet-state formation.^182^^,^^184^ Moreover, fluorescence lifetime in BODIPY-derived systems can be modulated by the local environment. Solvent polarity, aggregation, and biomolecular interactions influence nonradiative decay pathways, whereas pH-responsive functionalization (e.g., protonation of amine or phenolic groups) can induce structural changes that alter fluorescence behavior in biological settings.^185^^–^^187^ Together, these features demonstrate how structural design and environmental interactions govern fluorescence lifetime in BODIPY-based NIR fluorophores.

Alexa Fluor, ATTO, CF, and DyLight dyes provide complementary options for NIR FLI. Table 1 reports lifetimes of ∼0.4 to 1.5 ns for selected Alexa Fluor dyes,^33^^,^^37^^,^^159^^,^^188^^–^^197^ 0.5 to 3.3 ns for ATTO dyes,^38^^–^^45^ 0.4 to 3.2 ns for CF dyes,^46^^,^^48^^,^^49^^,^^51^^–^^53^^,^^198^^–^^203^ and 0.57 to 1.0 ns for the listed DyLight dyes.^64^^,^^66^^,^^68^^,^^204^ These measurements identify potential sources of temporal contrast, but their differing measurement conditions preclude a general ranking of dye performance. Brightness and photostability support photon collection, whereas lifetime reproducibility must be established for the formulation and environment being imaged.^205^^,^^206^

**Table 1:** Organic exogenous fluorophores. NS stands for not specified in the checked source.

Fluorophore	$\lambda_{\mathrm{abs}/\mathrm{ex}}$ (nm)	$\lambda_{\mathrm{em}}$ (nm)	ε $\left(M^{-1}\;{\mathrm{cm}}^{-1}\right)$	QY (%)	τ (ns)	Formulation/binding state	Measurement environment	Measurement modality
(Alexa Fluor) AF 647	650^33^	665^33^	270,000^33^	33^33^	1.0;^33^ 1.08;^34^ 1.12^34^	Free succinimidyl ester, unbound;^33^ AF647 Maleimide (1.08 ns);^34^ AF647 – C2 Maleimide (1.12 ns)^34^	PBS, 22°C (QY);^33^ $H_{2}O$, 22°C (τ);^33^ labeling buffer 1 (50 mM Tris-HCl pH7.4 to 8.0, 50 mM KCl, 5% glycerol)^34^	TCSPC FLI (confocal ALEX microscope)^34^
AF 660^33^	663	690	132,000	37	1.2	Free succinimidyl ester; unbound	PBS, 22°C (QY); pH 7.5 buffer, 22°C (τ)	manufacturer
AF 680^33^	679	702	183,000	36	1.2	Free succinimidyl ester; unbound	PBS, 22°C (QY); $H_{2}O$, 22°C (τ)	manufacturer
AF 700	702^35^	723^35^	205,000^35^	25^33^	1.0^33^	Free succinimidyl ester; unbound^33^	PBS, 22°C (QY); $H_{2}O$, 22°C (τ)^33^	manufacturer
AF 750	749^33^	775^33^	290,000^33^	12^33^	0.70;^33^ 0.61;^36^ 1.19^36^	Free succinimidyl ester; unbound^33^^,^^36^	PBS, 22°C (QY);^33^ $H_{2}O$, 22°C (0.70 ns);^33^ $H_{2}O$ (0.61 ns) and DMSO (1.19 ns)^36^	Fiber-scanning time-domain TCSPC-FLI^36^
AF 790^37^	784	814	260,000	4	0.4	Reactive amine-labeling dye	5 μM AF790 in a tube embedded in 1% Intralipid/50-ppm India-ink tissue phantom	Spectral/time-domain fluorescence mediated tomography
ATTO 655^38^	663	680	125,000	30	1.87; 3.31	Free dye	$H_{2}O$ (1.87 ns); EtOH (3.31 ns)	Manufacturer
ATTO 665^39^	662	680	160,000	60	2.9	Free dye	NS	Manufacturer
ATTO 678 H^40^	678	708	150,000	20	1.5	Free dye	NS	Manufacturer
ATTO 680^41^	680	700	125,000	30	1.69; 3.04	Free dye	$H_{2}O$ (1.69 ns); EtOH (3.04 ns)	Manufacturer
ATTO 700^42^	700	719	120,000	25	1.6	Free dye	NS	Manufacturer
ATTO 725^43^	728	751	120,000	10	0.5	Free dye	NS	Mmanufacturer
ATTO 740^44^	743	763	120,000	10	0.6	Free dye	NS	Manufacturer
ATTO Oxa12^45^	662	681	125,000	30	1.8	Free dye	NS	Manufacturer
CF647	650^46^	665^46^	240,000^46^	NS	1.07^47^	Free dye^47^	PBS	Manufacturer
CF660R^48^	662	682	100,000	NS	3.24	Free dye	PBS	Manufacturer
CF680R	680^49^	701^49^	140,000^49^	NS	1.22^50^	Free dye	PBS	Manufacturer
CF680^51^	681	698	210,000	NS	1.23	Free dye	PBS	Manufacturer
CF750^52^	755	777	250,000	NS	0.58	Free dye	PBS	Manufacturer
CF790^53^	784	806	210,000	NS	0.39	Free dye	PBS	Manufacturer
Cy5	647^54^	665^54^	270,000^54^	26^54^	1.5, 1.96;^55^ 1.03, 1.23, 2^54^	Free dye;^55^ Cy5-dCTP^54^	Ethanol (1.5 ns), ethyl iodide-doped ethanol solution (1.96 ns);^55^ Cy5-dCTP in aqueous solution (1.03 ns), on dry surface (1.23 ns), embedded in film (2 ns)^54^	Spectral/time-resolved single-molecule TCSPC^54^^,^^55^
Cy5.5	678^56^	695^56^	190,000^56^	28;40^57^	1, 1.6^58^	PBS (QY: 28%), Ethanol (QY: 40%)^57^	*In vivo* kidney (1.6 ns)^58^	Time-domain eXplore Optix FLI^58^
Cy7	756^59^	779^59^	200,000^59^	30^60^	1^61^	Solid Cy7 inclusion^61^	10 μM Cy7 inclusion embedded in an Intralipid phantom^61^	Time-domain fluorescence eXplore Optix-MX2 system^61^
Cypate^17^	750	850	224,000	35	0.46; 0.87	Free dye	MeOH (0.46 ns); DMSO (0.87 ns); room temperature	TCSPC fluorescence with R928P detector
DTTCI	750^17^	850^17^	212,000^17^	38^62^	1.07;^17^ 1.49;^17^ 1.18^63^	Free dye^17^^,^^63^	MeOH (1.07 ns); DMSO (1.49 ns), room temperature^17^^,^^63^	TCSPC fluorescence with R928P detector^17^ PMT-based frequency-domain fluorescence tomography^63^
DyLight 649	652^64^	672^64^	250,000^65^	18^64^	1^64^	DyLight-649 anti-mouse IgG bound to surface-immobilized mouse IgG^64^	Silver fractal substrates (glass/plastic/silicon), covered with 50 mM sodium-phosphate buffer, pH 7.4; conjugate 33 nM^64^	Confocal TCSPC-FLIM^64^
DyLight 750	752^66^	778^66^	210,000^65^	6^67^	0.712^66^	DyLight750–HER2-specific Affibody (${\mathrm{His}}_{6}\text{-}Z_{\mathrm{HER}2}:\mathrm{GS}\text{-}\mathrm{Cys}$), 1:1 dye/protein^66^	Probe bound to HER2-positive SK-BR-3 cells in PBS; 0.712 ns agrees with the uninjected probe (∼0.710 ns)^66^	TCSPC-FLIM with Olympus FV1000 inverted laser-scanning two-photon microscope^66^
DyLight 755	754^68^	776^68^	220,000^68^	11.9^68^	0.57^69^	DyLight755-conjugated iron-loaded transferrin^69^	Fixed T47D breast-cancer cells after 2-h transferrin incubation and washing^69^	Wide-field time-gated FLI/FRET with ICCD detector^69^
FLUX 647^70^	650	666	270,000	26	1.1	Free dye	NS	NS
FLUX 660^71^	663	680	210,000	19	0.9	Free dye	NS	NS
FLUX 680^72^	674	695	250,000	18	0.9	Free dye	NS	NS
FROG-1-${\mathrm{NH}}_{4}$ (5a)^73^	718	748	117,000	18	0.66	Free dye	DMSO, room temperature; absorbance <0.15 at 700 nm	TCSPC FLIM with MicroTime200 confocal microscope
FROG-2-${\mathrm{NH}}_{4}$ (5b)^73^	718	748	107,000	17	0.41	Free dye	DMSO, room temperature; absorbance <0.15 at 700 nm	TCSPC FLIM with MicroTime200 confocal microscope
HiLyte 647^74^	650	675	250,000	33	1	HiLyte Fluor 647 acid; free dye	NS	NS
PromoFluor 680	690^75^	710^76^	140,000^75^	40.5^75^	0.405^75^	Free dye^75^	EtOH^75^	NS
PPC-${\mathrm{BF}}_{2}$^77^	750	850	530,000 to 570,000	20	3.89	Free dye and albumin-bound dye	Albumin/$H_{2}O$	TCSPC FLI
PPC-${\mathrm{BPh}}_{2}$^77^	810	900	500,000 to 530,000	5	3.35; 2.95	Free dye and albumin-bound dye	DMSO (3.35 ns); albumin/$H_{2}O$ (2.95 ns)	TCSPC FLI
Seta-670-NHS^78^	667	688	180,000	7; 27	0.42; 2.43	PB 7.4 (QY:7%); BSA-bound (QY:27%)	PBS (0.42 ns); BSA solution (2.43 ns)	NS
Seta-660-di-NHS^79^	661	672	220,000	11; 49	0.9	PB 7.4 (QY:11%); IgG (QY:49%)	PB, pH 7.4	NS
SeTau-647-di-NHS^80^	650	694	200,000	65; 58	3.2	PB 7.4 (QY:58%); IgG (QY:65%)	PB, pH 7.4	NS
SeTau-665-NHS^81^	664	712	161,000	53; 46	3.1	PB 7.4 (QY:53%); IgG (QY:46%)	PB, pH 7.4	NS
SeTau-680-NHS^82^	683	705	295,000	24; 30	2.9; 3.1	PB 7.4 (QY:24%, 2.9 ns); IgG (QY:30%, 3.1 ns)	NS	NS
SiR650-BG^83^	652	674	116,000	37	3	Benzylguanine-functionalized Si-rhodamine probe	NS	NS
IRDye 700DX	689^84^	700^84^	170,000^84^	12^85^	3.56^86^	Panitumumab–IR700 conjugate (Pan-IR700) (τ);^86^ unbound, BSA-bound, and blood states (QY)^85^	Pan-IR700 in PBS at 2.5, 5, 20, and 40 μg/mL, room temperature (τ);^86^ unbound (QY)^85^	Time-domain FLI eXplore Optix-MX2 system^86^
IRDye 800CW^87^	774	789	242,000	9	0.435; 1.1	Free dye	PBS (0.435 ns); DMSO (1.1 ns)	Time domain mesoscopic fluorescence molecular tomography with TCSPC SPAD-array detection
Methylene blue^88^	668	685	74,000	2;4	0.3 to 0.6	Free dye	$H_{2}O$, EtOH, and binary solvent mixtures; conditions are solvent-series specific	Time-resolved fluorescence spectroscopy with Perkin Elmer LS 55 fluorescence spectrometer
Indocyanine Green (ICG)	780^89^	820^89^	260,000^89^	14^89^	0.52;^17^ 0.51;^17^ 0.97^17^	Free ICG^17^^,^^89^	$H_{2}O$ (0.52 ns); MeOH (0.51 ns); DMSO (0.97 ns); room temperature^17^	TCSPC fluorescence with R928P detector^17^
Cytalux (OTL-38)	776^90^	796^90^	NS	NS	1.95^90^	Folate-analog–S0456 conjugate; intact targeted agent;^90^ OTL-38 conjugate in solid phantom^90^	OTL-38-mimicking lung solid phantom^90^	Macroscopic time-domain FLI (TauVIVO)^90^^,^^91^
ZW800-1A	772 to 792^92^	788 to 814^92^	246,000 to 256,000^92^	13.5 to 26.5^92^	0.64;^93^ 0.98;^93^ 0.84^93^	Intact zwitterionic dye; tissue-bound/vascular states^93^	Mouse brain: blood vessel (0.64 ns); CAA (0.98 ns); amyloid plaques (0.84 ns)^93^	Two-photon TCSPC-FLIM^93^
ZW800-1C	779^92^^,^^93^	772 to 798^92^^,^^93^	76,000 to 225,000^92^^,^^93^	NS	0.84;^93^ 1.14;^93^ 1.27;^93^ 1.24^93^	Intact zwitterionic dye; bound to pathological protein aggregates/tissue structures^93^	Mouse brain: blood vessel (0.84 ns); NFTs (1.14 ns); CAA (1.27 ns); amyloid plaques (1.24 ns)^93^	Two-photon TCSPC-FLIM^93^

For multiplexing, the relevant advantage is reproducible separation within the selected probe set. CF660R and CF680R, for example, have reported lifetimes of 3.24 and 1.22 ns in PBS, respectively,^48^^,^^49^^,^^51^ making them candidates for evaluation as distinguishable lifetime labels. Their performance in mixed samples or tissue nevertheless depends on photon counts, relative signal contributions, background, and changes following conjugation or binding. Environmental shifts can be substantial, and Alexa Fluor 750 has reported lifetimes of 0.61 ns in water and 1.19 ns in DMSO.^16^^,^^33^^,^^36^^,^^207^^,^^208^ Such variation complicates assignment from a fixed reference lifetime and motivates characterization under the intended biological conditions.

The available measurements therefore support condition-specific selection of candidate lifetime labels, whereas quantitative environmental sensing requires a separately established calibration and assessment of selectivity. For FLI-FRET, the corresponding requirement is a reproducible donor-only decay for the relevant conjugate and environment, allowing transfer-induced shortening to be distinguished from unrelated changes in photophysics.

Beyond these established fluorophores, recent carbocyanine derivatives like CJ215 push emission beyond 800 nm and into the short-wave infrared (SWIR) window, achieving tumor-to-background ratios up to 100 without the need for conjugated targeting ligands.^209^^,^^210^ This selectivity arises from CJ215’s affinity for apoptotic and ferroptotic cells via phosphatidylserine binding, allowing tumor-specific imaging *in vivo* and extending fluorescence guidance into postoperative monitoring.^210^ Representative long-lifetime organic NIR fluorophores are summarized in Table 2.

**Table 2:** Long lifetime organic NIR fluorophores.

Fluorophore	$\lambda_{\mathrm{abs}/\mathrm{ex}}$ (nm)	$\lambda_{\mathrm{em}}$ (nm)	ε $\left(M^{-1}\;{\mathrm{cm}}^{-1}\right)$	QY (%)	τ (ns)	Formulation/binding state	Measurement environment	Measurement modality
Diphenyldithienyl aza-BODIPY (DPDTAB)^211^	717	739	114,500	47	3.71	Free dye	${\mathrm{CH}}_{2}{\mathrm{Cl}}_{2}$	TCSPC fluorescence lifetime spectroscopy (Edinburgh OB 920)
	720	741	120,700	44	3.70	Free dye	Toluene	
	717	738	120,500	46	3.22	Free dye	Tetrahydrofuran (THF)	
	710	732	108,600	46	3.60	Free dye	MeCN	
	728	746	118,300	20	3.40	Free dye	DMSO	
Tetrathienyl aza-BODIPY (TTAB)^211^	732	757	103,100	12	2.23	Free dye	Acetonitrile	TCSPC fluorescence lifetime spectroscopy (Edinburgh OB 920)
	740	764	109,900	12	2.36	Free dye	${\mathrm{CH}}_{2}{\mathrm{Cl}}_{2}$	
	743	768	107,500	10	2.21	Free dye	Toluene	
	740	763	107,300	12	1.95	Free dye	THF	
	752	775	108,000	13	2.34	Free dye	DMSO	
thienoWaza^212^	718	739	78,600	6	1.9	Free dye	DMSO	TCSPC fluorescence lifetime spectroscopy
	716	728	68,300	5	2.9	Dye with BSA	PBS containing 5% BSA	
thienoWaza-1^212^	720	739	88,750	5	3.0	Free dye	DMSO	TCSPC fluorescence lifetime spectroscopy
	709	723	41,750	5	1.1	Free dye	PBS	
	716	728	69,500	10	2.8	Dye with BSA	PBS containing 5% BSA	
thienoWaza-2^212^	722	740	115,000	26	3.2	Free dye	DMSO	TCSPC fluorescence lifetime spectroscopy
	716	725	43,000	3	1.3	Free dye	PBS	
	716	728	68,250	5	2.9	Dye with BSA	PBS containing 5% BSA	
thienoWaza-BC1^212^	724	740	108,300	20	2.9	Free dye	DMSO	TCSPC fluorescence lifetime spectroscopy
	715	734	105,500	10	1.5	Free dye	PBS	
	715	733	107,600	10	1.8	Dye with BSA	PBS containing 5% BSA	
	715	729	103,800	11	1.6	Dye with BSA	PBS containing 10% BSA	
aza-FL^213^	734	741	229,000	66	4.3	Free dye	THF^a^	TCSPC (Horiba DeltaHub)
aza-CZ^213^	791	800	237,000	49	2.3	Free dye	THF^a^	TCSPC (Horiba DeltaHub)
aza-OHCl^213^	763	776	161,000	16	1.7	Free dye	THF^a^	TCSPC (Horiba DeltaHub)
TPAB (tetraphenyl aza-BODIPY)^213^	652	677	79,000	34	0.9	Free dye	THF^a^	TCSPC (Horiba DeltaHub)
$\mathrm{ADP}(\mathrm{CA}{)}_{2}$ ^214^	696	720	NS	0.56	2.3 to 3 ns^b^	Free dye	Water	Time-resolved detection within a focused ultrasound activation volume
	742	720	NS	7.45		Free dye	DMSO	
	709	720	NS	14.51		Free dye	Toluene	
	678	720	NS	10.51		Free dye	Dioxane	
	678	720	NS	12.4		Free dye	Dichloroethane	
	678	720	NS	7.93		Free dye	Ethylene, glycerol	
	678	720	NS	16.22		Free dye	Glycerol	
PPCy 4a	757^127^	779^127^	205,000^128^	59^128^	4.02^127^	Free dye^127^	DMSO (λ, τ);^127^ ${\mathrm{CHCl}}_{3}$ (ε, QY)^128^	TCSPC FLI^127^
	773^127^	830^127^	205,000^128^	59^128^	3.89^127^	Albumin-bound dye^127^	Albumin/$H_{2}O$ (λ, τ);^127^ ${\mathrm{CHCl}}_{3}$ (ε, QY)^128^	TCSPC FLI^127^
PPCy 4a′	824^127^	840^127^	256,000^128^	53^128^	3.35^127^	Free dye^127^	DMSO (λ, τ);^127^ ${\mathrm{CHCl}}_{3}$ (ε, QY)^128^	TCSPC FLI^127^
	773^127^	830^127^	256,000^128^	53^128^	2.95^127^	Albumin-bound dye^127^	Albumin/$H_{2}O$ (λ, τ);^127^ ${\mathrm{CHCl}}_{3}$ (ε, QY)^128^	TCSPC FLI^127^

a for THF only; aqueous data not reported.

b represents lifetime increases as solvent polarity decreases.

Building on the established dye families described above, recent efforts have focused on engineering donor–acceptor–donor (D–A–D) architectures, conformationally restricted scaffolds, heteroatom substitution, and extension of conjugation to shift emission into the NIR and SWIR regions while modulating nonradiative decay pathways, thereby improving performance.^170^^,^^215^ For example, oxonol dyes incorporating 1,6-diheterophenalene-2,5-dione (DHPD) end groups demonstrate that structural rigidification suppresses cis–trans isomerization and improves fluorescence efficiency. FROG-1 and FROG-2 exhibit NIR absorption/emission (∼718/748  nm), with lifetimes of 0.66 and 0.41 ns, quantum yields of 0.18 and 0.17 (relative to HITCI), and molar absorptivities of $1.17\times 10^{5}$ and $1.07\times 10^{5}\;M^{-1}\;{\mathrm{cm}}^{-1}$ in DMSO, as shown in Fig. 3.^73^ Despite similar optical properties, small structural differences lead to distinct biological behavior, with FROG-2 showing enhanced tumor uptake and FROG-1 undergoing rapid renal clearance. These results highlight the role of structure-guided design in decoupling photophysics from biodistribution for advanced FLI applications.^73^

**Fig. 3:**
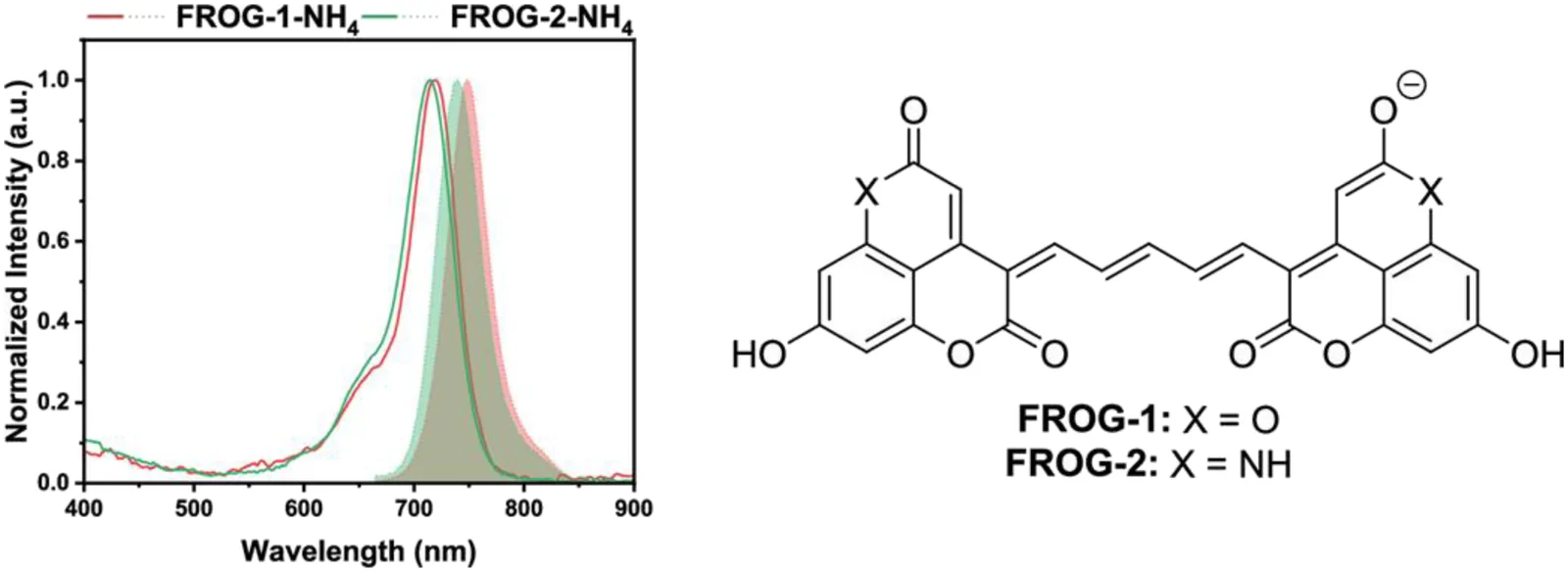
Absorption and emission spectra in DMSO and chemical structures of FROG-1-${\mathrm{NH}}_{4}$ (5a) and FROG-2-${\mathrm{NH}}_{4}$ (5b). Reproduced with permission from Ref. 73, licensed under CC BY-NC-ND 4.0.

Beyond their effects on cellular uptake and biodistribution, structure-guided modifications have also been used to expand the lifetime contrast available for NIR FLI. Conventional heptamethine cyanine dyes commonly exhibit lifetimes below 1 ns, which can limit their discrimination from endogenous tissue AF in lifetime-based imaging. Consequently, the development of long-lifetime NIR fluorophores has become an important objective for FLI. As summarized in Table 2, longer lifetimes expand the accessible temporal contrast and enable more reliable separation of probe signals from short-lived background fluorescence.

In contrast to flexible polymethine cyanines, several recently developed NIR fluorophores incorporate structurally rigid chromophore frameworks that restrict intramolecular rotation and reduce nonradiative decay. For example, pyrrolopyrrole-based cyanine derivatives exhibit fluorescence lifetimes of ∼2 to 4 ns^127^^,^^128^ while maintaining strong absorption and emission within the NIR spectral window. Similarly, aza-BODIPY-derived fluorophores and related thieno-fused analogs display lifetimes approaching ∼4  ns in both organic solvents and physiologically relevant environments while preserving high molar absorptivity and quantum yields.^211^^–^^214^ These extended lifetimes arise primarily from the rigid and planar chromophore frameworks shown in Fig. 4(a)^213^ and 4(b),^211^ which limit torsional relaxation and suppress internal conversion, thereby favoring radiative decay and increasing the excited-state lifetime. These design strategies highlight how structural control of molecular rigidity and conjugation can extend fluorescence lifetimes in the NIR region and provide a framework for the development of next-generation probes for biological and clinical imaging applications shown in Fig. 4(c).

**Fig. 4:**
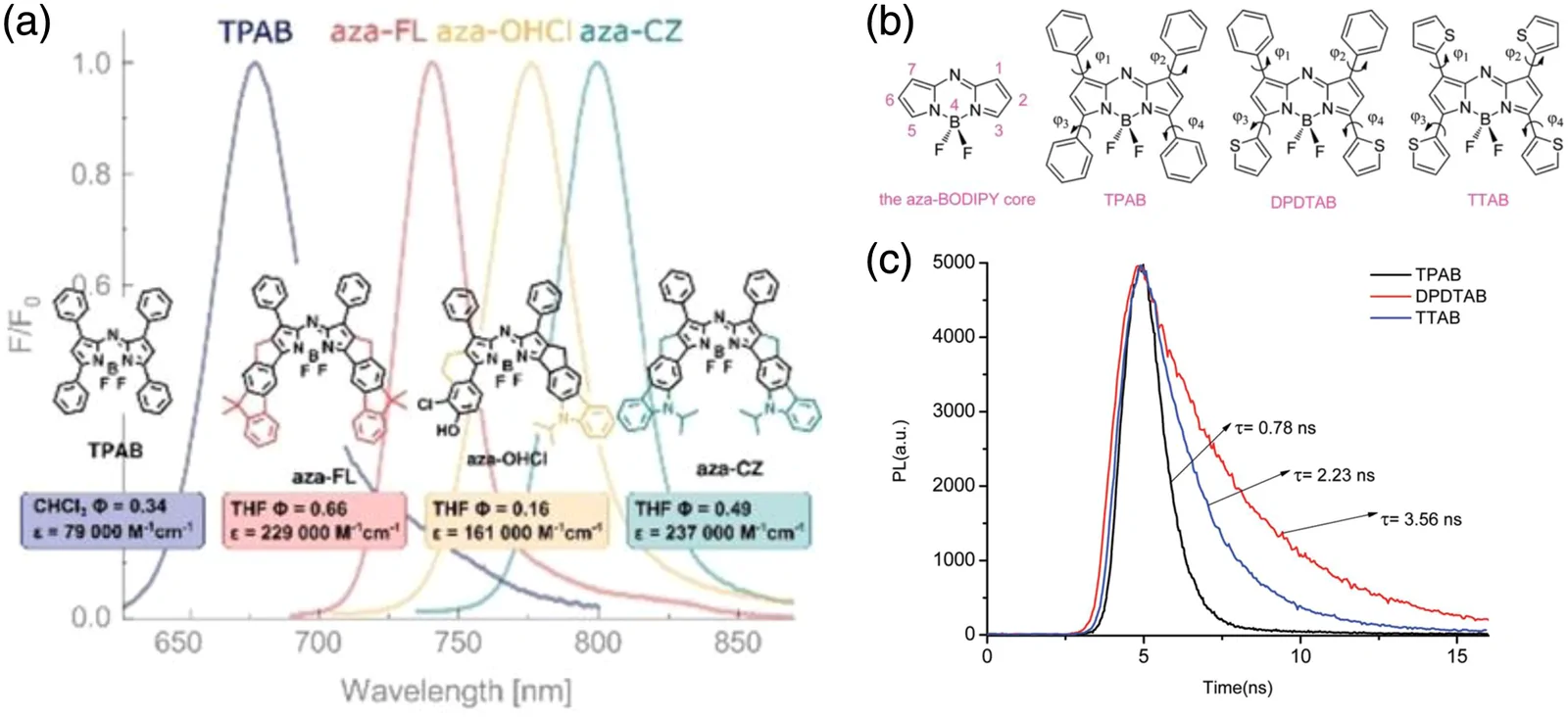
(a) Chemical structures and representative photophysical properties of fluorene- and carbazole-fused aza-BODIPY fluorophores exhibiting bathochromically shifted NIR absorption and emission with high brightness and photostability. Adapted with permission from Ref. 213, licensed under CC BY. (b) Structures of the aza-BODIPY core, TPAB, DPDTAB, and TTAB, where $\phi_{1}\text{–}\phi_{4}$ denote torsion angles between the aromatic rings and the central chromophore plane. (c) Fluorescence lifetime decay curves of TPAB (black), DPDTAB (red), and TTAB (blue) in acetonitrile. Panels (b) and (c) are reproduced with permission from Ref. 211.

Collectively, these advances highlight the growing importance of NIR-emitting and, more recently, SWIR-emitting organic dyes as adaptable tools for lifetime-based imaging, with emerging opportunities in areas such as metabolic sensing, microenvironment mapping, and image-guided surgery.

### 3.2 Exogenous Fluorophores: Fluorescent Proteins

Beyond synthetic small-molecule dyes, far-red and NIR fluorescent proteins (FPs) provide genetically encoded probes for cellular and tissue imaging. These proteins originate from several distinct scaffolds. Established iRFPs and several miRFP variants are derived from bacterial phytochromes, whereas miRFP670 nano was engineered from a cyanobacteriochrome and smURFP from an allophycocyanin α-subunit. These probes incorporate biliverdin IXα (BV) as their chromophore but differ in structure, oligomeric state, and cellular performance.^216^^–^^218^

BV is a naturally occurring product of heme metabolism, and efficient incorporation of endogenous BV enables selected variants to fluoresce in mammalian cells without supplementation. However, endogenous availability does not ensure equivalent fluorescence across proteins or biological systems; cellular brightness depends on protein expression, chromophore availability, and incorporation efficiency. Photostability, oligomeric state, and cytotoxicity should likewise be evaluated for the intended application.^217^^–^^219^ The proteins summarized in Table 3 have emission maxima concentrated around 670 to 720 nm and reported sub-nanosecond to nanosecond fluorescence lifetimes under the specified measurement conditions.

**Table 3:** Fluorescent proteins, emiRFP670 photophysical properties are equivalent to those of the miRFP670 parent.

Fluorophore	$\lambda_{\mathrm{abs}/\mathrm{ex}}$ (nm)	$\lambda_{\mathrm{em}}$ (nm)	ε $\left(M^{-1}\;{\mathrm{cm}}^{-1}\right)$	QY (%)	τ (ns)	Formulation/binding state	Measurement environment	Measurement modality
miRFP670	642^220^	670^220^	87,400^220^	14.0^220^	1.22^221^	BV-bound miRFP670 expressed in *E. coli*^221^	Layer of living miRFP670-expressing *E. coli*^221^	Time-domain FLIM^221^
emiRFP670	642^216^	670^216^	87,400^216^	14^216^	1.53^219^	Purified BV-bound emiRFP670^219^	PBS, pH 7.4^219^	Frequency-domain ChronosDFD system^219^
miRFP670-2	643^216^	670^216^	103,000^216^	13.6^216^	1.42^219^	Purified BV-bound miRFP670-2^219^	PBS, pH 7.4^219^	Frequency-domain ChronosDFD system^219^
iRFP670	643^220^	670^220^	114,000^220^	12.2^220^	0.93^222^	BV-bound iRFP670 genetically expressed in MTLn3 tumor cells^222^	Living mouse bearing an iRFP670-expressing MTLn3 mammary tumor^222^	Time-domain FLI^222^
miRFP680	661^216^	680^216^	94,000^216^	14.5^216^	1.67^219^	Purified BV-bound miRFP680^219^	PBS, pH 7.4^219^	Frequency-domain ChronosDFD system^219^
iRFP682^222^	663	682	90,000	11.2	0.93	BV-bound iRFP682 genetically expressed in MTLn3 tumor cells	Living mouse bearing an iRFP682-expressing MTLn3 mammary tumor	Time-domain FLI
iRFP702	673^220^	702^220^	93,000^220^	8.2^220^	0.78^222^	BV-bound iRFP702 genetically expressed in MTLn3 tumor cells^222^	Living mouse bearing an iRFP702-expressing MTLn3 mammary tumor^222^	Time-domain FLI^222^
miRFP703	674^221^	703^221^	90,900^221^	8.6^221^	0.64^221^	BV-bound miRFP703 expressed in *E. coli*; unphotoconverted ground state^221^	Layer of living miRFP703-expressing *E. coli*; pH and temperature NS^221^	Time-domain FLIM^221^
iRFP713	690^220^	713^220^	98,000^220^	6.3^220^	0.68^222^	BV-bound iRFP713 genetically expressed in MTLn3 tumor cells^222^	Living mouse bearing an iRFP713-expressing MTLn3 mammary tumor^222^	Time-domain FLI^222^
iRFP720	702^220^	720^220^	96,000^220^	6.0^220^	0.68^222^	BV-bound iRFP720 genetically expressed in MTLn3 tumor cells^222^	Living mouse bearing an iRFP720-expressing MTLn3 mammary tumor^222^	Time-domain FLI^222^
miRFP720	702^221^	720^221^	98,000^221^	6.1^221^	0.65^221^	BV-bound miRFP720 expressed in *E. coli*; unphotoconverted ground state^221^	Layer of living miRFP720-expressing *E. coli*; pH and temperature NS^221^	Time-domain FLIM^221^
smURFP	642^223^	672^223^	180,000^223^	18.0^223^	1.40^223^	Purified homodimeric smURFP bound to BV^223^	PBS^223^	Digital frequency-domain lifetime ChronosDFD system^223^
mIFP	683^220^	705^220^	65,900^220^	6.9^220^	0.80^219^	Purified BV-bound mIFP^219^	PBS, pH 7.4^219^	Frequency-domain ChronosDFD system^219^
SNIFP	697^224^	720^224^	149,000^224^	2.2^224^	0.63^219^	Purified BV-bound SNIFP^219^	PBS, pH 7.4^219^	Frequency-domain ChronosDFD system^219^

Genetic encoding enables cell-specific expression and, for suitable variants, fusion to proteins of interest. For lifetime multiplexing, probe selection should additionally establish that decay signatures remain distinguishable in the intended cellular environment. In our laboratory, emiRFP670 and miRFP680 exhibited distinguishable fluorescence decays following expression in AU565 HER2-positive breast cancer cells, supporting their potential as a lifetime-multiplexing pair under the tested conditions^225^ [Figs. 5(b) and 5(c)]. A representative protein structure is shown in Fig. 5(a). Extension to other fusion constructs, cell types, or tissue settings requires confirmation of expression, biological compatibility, and lifetime separation.

**Fig. 5:**
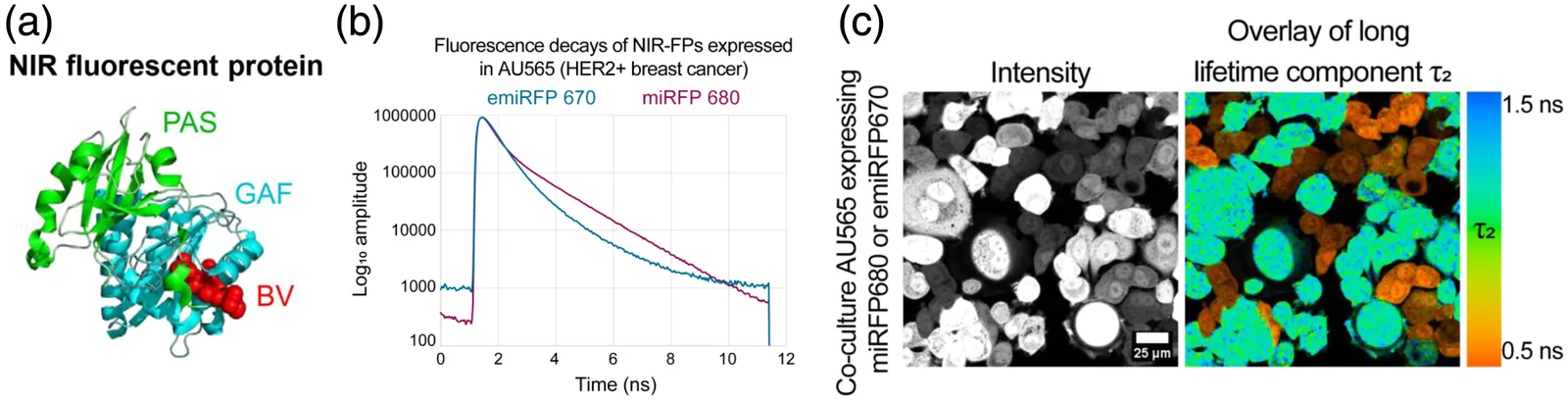
(a) Two-domain NIR structure engineered from BphP. Reproduced with permission from Ref. 12. (b) Fluorescence decays measured from emiRFP670 (blue) and miRFP680 (red) following expression in AU565 HER2+ breast cancer cells.^225^ (c) TCSPC image captured on Becker & Hickl’s HPM-100-50 hybrid detector showing a co-culture of AU565 cells expressing emiRFP670 or miRFP680.^225^ The fluorescence decays of this pair of NIR-FPs enable them to be clearly distinguished via lifetime multiplexing.

### 3.3 Exogenous Fluorophores: Inorganic Fluorophores

Inorganic NIR fluorophores provide exceptional photostability, large Stokes shifts, and tunable lifetimes beyond those of organic dyes. Fluorophores with characterized lifetimes are summarized in Table 4. Among these, semiconductor quantum dots (QDs) and metal-complex molecular probes are the most studied for FLI. NIR-emitting QDs such as CdTe, CdSe, PbS, ${\mathrm{CuInS}}_{2}$, and ${\mathrm{Ag}}_{2}S$ exhibit broad absorption and narrow emission peaks covering 650 to 950 nm. Their quantum yields often exceed 50% with lifetimes of tens to hundreds of nanoseconds, enabling time-gated and multiplexed lifetime imaging.^231^^,^^232^^,^^235^^,^^237^^–^^241^ For example, QD705 effectively suppressed short-lived AF through time-gated detection, enhancing *in vivo* contrast.^231^ Distinct QDs can also be separated spectro-temporally for multiplexed and FRET-based imaging.^228^^,^^242^^–^^244^ However, potential toxicity from ${\mathrm{Cd}}^{2+}$ or ${\mathrm{Pb}}^{2+}$ leakage and fluorescence blinking remain challenges.^245^ Surface passivation and alloyed or Ag/Cu-based cores have improved biocompatibility while preserving long-lived, bright NIR emission.^232^^,^^235^^,^^237^

**Table 4:** Quantum dots and inorganic metal-complex fluorophores.

Class	Fluorophore	Composition	$\lambda_{\mathrm{ex}}$ (nm)	$\lambda_{\mathrm{em}}$ (nm)	ε $\left(M^{-1}\;{\mathrm{cm}}^{-1}\right)$	QY (%)	τ (ns)	Measurement environment	Measurement modality
Quantum Dots	QD655	CdSe/ZnS^226^	655^226^	<670 ^226^	N/A	16.9 to 18^227^	13 to 41^228^^,^^229^	Free QD655 in PBS, pH 7.0;^228^ in PBS, pH 7.0;^228^ in water and individual particles^229^	TCSPC time-resolved luminescence FluoroMax 4 system;^228^ confocal single-particle FLIM^229^
	QD705	CdSeTe/ZnS^230^	<700 ^230^	705^230^	N/A	>60 ^230^	>20 ^230^	Subcutaneous QD705 injections in mice^231^	Wide-field time-gated fluorescence imaging^231^
	QD750	CdTeSe/ZnS^232^	<740 ^232^	750^232^	N/A	N/A	12 to 31^232^	QD750–LS662 conjugate in pH 2 to 10 buffers;^232^ in 0.1 M NaCl titrated with HCl or NaOH;^232^	Time-resolved fluorescence-decay measurement^232^
	QD800	CdSeTe/ZnS^233^	<760 ^233^	800^233^	N/A	>26 ^233^	77.8^234^	L-histidine-capped QD800 dispersed in aqueous borate buffer following phase transfer^234^	Time-resolved photoluminescence-decay spectroscopy^234^
	—	ZnCuInSe/ZnS^235^	659^235^	800^235^	N/A	20 to 35^235^	$\tau_{\text{short}}$: 25 to 60,^235^ $\tau_{\text{long}}$: 134 to 177^235^	Ligand-exchanged QDs in water: $\tau_{\text{short}}=25\;\mathrm{ns}$^235^ and $\tau_{\text{long}}=134\;\mathrm{ns}$;^235^ QDs in HeLa-cell cytoplasm 24 h after electroporation:^235^ $\tau_{\text{short}}=60\;\mathrm{ns}$^235^ and $\tau_{\mathrm{long}}=177\;\mathrm{ns}$^235^	Wide-field time-gated fluorescence-decay imaging^235^
Metal Complex Probes	Zn-OMeNc	Zinc naphthalocyanine^236^	794^236^	804^236^	N/A	N/A	1.7^236^	Free Zn-OMeNc in DMSO^236^	Solution-phase TCSPC fluorescence-decay spectroscopy^236^
	$\mathrm{Zn}\text{-}{\mathrm{NMe}}_{2}\mathrm{Nc}$	Zinc naphthalocyanine^236^	800^236^	811^236^	N/A	N/A	0.86^236^	Free $\mathrm{Zn}\text{-}{\mathrm{NMe}}_{2}\mathrm{Nc}$ in DMSO^236^	Solution-phase TCSPC fluorescence-decay spectroscopy^236^
	$\mathrm{Zn}\text{-}{\mathrm{NMe}}_{3}\mathrm{Nc}$	Zinc naphthalocyanine^236^	732 to 781^236^	801 to 804^236^	N/A	N/A	2.4 to 5.69^236^	Free $\mathrm{Zn}\text{-}{\mathrm{NMe}}_{3}\mathrm{Nc}$ in water for 2.4 ns;^236^ free $\mathrm{Zn}\text{-}{\mathrm{NMe}}_{3}\mathrm{Nc}$ in DMSO for 5.69 ns;^236^	Solution-phase TCSPC fluorescence-decay spectroscopy^236^
	IRDye 700DX	Silicon–phthalocyanine derivative^a^^86^	670^86^	700^86^	N/A	N/A	2.85 to 3.56^86^	Panitumumab–IR700 in PBS at 2.5 to $40\;\mu g\;{\mathrm{mL}}^{-1}$:^86^ mean lifetime 3.56±0.081 ns;^86^ A431-cell pellets after 24-h incubation with Pan–IR700^86^ and $30\;J\;{\mathrm{cm}}^{-2}$ NIR photoimmunotherapy:^86^ 2.85 ns^86^	Time-domain fluorescence-lifetime imaging using an eXplore Optix–MX2 system^86^

For QDs, $\lambda_{\mathrm{ex}}$ denotes the upper bound of the broadband excitation range; QD excitation is effective at all wavelengths below this value.

a IRDye 700DX is a silicon-phthalocyanine conjugated dye and appears in Table 1 under organic exogenous fluorophores.

Metal-complex NIR fluorophores, such as metallophthalocyanines (ZnPc, AlPc, SiPc) and related chlorins, feature strong Q-band absorption (630 to 690 nm) and emission near 700 to 800 nm.^246^^,^^247^ These molecules exhibit mono-exponential lifetimes of 3 to 7 ns, depending on solvent polarity and aggregation state.^246^^,^^248^^,^^249^ Silicon phthalocyanines show particularly long-lived fluorescence (6.7 to 7.3 ns) and high photostability, making them promising for FLIM.^246^^,^^248^ Lifetime imaging of metallated phthalocyanines also reveals microenvironmental effects such as aggregation or protein binding, with ZnPc-albumin conjugates maintaining intrinsic nanosecond lifetimes while liposomal formulations display aggregation-induced quenching.^249^^–^^251^

### 3.4 Endogenous Fluorophores and NIR Autofluorescence

Endogenous fluorophores are native biomolecules that provide intrinsic fluorescence contrast without the administration of an external probe. Compared with the broad range of exogenous fluorophores available for NIR imaging, relatively few endogenous fluorophores have well-characterized emission and fluorescence lifetime signatures within the NIR spectral range, as most intrinsic fluorophores emit primarily in the ultraviolet (UV) or visible region. Table 5 summarizes representative endogenous fluorophores relevant to NIR FLI, including fluorophores investigated using NIR two-photon excitation and fluorophores with emission extending into the NIR spectral range.

**Table 5:** Endogenous fluorophores.

Fluorophore	$\lambda_{\mathrm{ex}}$ (nm)	$\lambda_{\mathrm{em}}$ (nm)	ε $\left(M^{-1}\;{\mathrm{cm}}^{-1}\right)$	τ (ns)	Measurement environment	Measurement modality
Melanin	740 to 760^94^^,^^95^	780 to 900^95^^,^^96^	265 to 583^97^	0.2 to 0.4^a^; 1.8 to 2.5^b^^94^^–^^96^	Human skin and hair;^94^ in vivo and ex vivo pigmented skin and melanoma specimens^95^	Multiphoton FLIM and multiphoton laser tomography;^94^^,^^95^ FLI ophthalmoscopy^96^
Protoporphyrin IX	635^98^	700^99^^,^^100^	160,000^98^^,^^100^	3;^99^6;^101^ 16^101^	Ex vivo human glioma tissue(3 ns);^99^ ALA-induced PpIX in living MAT-LyLu rat prostate adenocarcinoma cells (6 ns);^101^ PpIX in DMSO (16 ns)^101^	Wide-field time-domain FLIM;^99^ Two-photon TCSPC-FLIM and streak-camera lifetime spectroscopy^101^
NADH	740 to 800^102^^,^^103^	NS	NS	0.5^a^; 2.1^b^^102^	Physiological salt solution^102^	Multiphoton excited-fluorescence lifetime measurement;^102^ two-photon FLIM for in vivo cerebral NADH measurements^103^
Hemoglobin	700 to 800^102^^,^^104^	NS	602 to 2970^105^^,^^106^	0.3^a^; 1.2^b^^102^^,^^104^^,^^107^	In physiological salt solution^102^	Multiphoton excited-fluorescence microscopy with third-harmonicgeneration;^102^ time-resolved fluorescence spectroscopy;^107^ two-photon FLIM^104^
Bilirubin	1230	650 to 800^108^^,^^109^	152 at 590 nm^109^	0.5^a^, 2^b^;0.3^a^, 1.5^b^^108^	Human hepatocellular-carcinoma tissue (0.5^a^, 2^b^ ns); paired nontumorous liver tissue (0.3^a^, 1.5^b^ ns)^108^	Two-photon FLIM^108^
Lipofuscin	1060^110^	604 to 679^110^	NS	0.1 to 0.5^110^	Untreated and drug-treated cancer cells, tumor spheroids, organoids, and three-dimensional tumor slices;^110^ temperature maintained at 37°C and 5% ${\mathrm{CO}}_{2}$ during live-cell imaging^110^	Two-photon TCSPC-FLIM^110^
Riboflavin	NS	700 to 800^102^	6737 to 7200^111^^,^^112^	4^102^	Riboflavin in physiological salt solution^102^	Multiphoton excited-fluorescence measurement^102^

Superscript letters link lifetime values to their corresponding measurement environments. NS indicates not specified in the checked source. QY is not reported; values are generally <1% for most endogenous NIR emitters.

a indicates short lifetime components ($\tau_{1}$).

b indicates long lifetime components ($\tau_{2}$).

Melanin, the broadband pigment present in the skin, hair, and retinal pigment epithelium exhibits an exceptionally low quantum yield but a detectable emission tail extending into NIR (800 to 900 nm).^97^^,^^252^^–^^255^ Time-resolved and adaptive optics ophthalmoscopy studies have confirmed melanin as the predominant endogenous source of NIR fluorescence.^256^^,^^257^ Fluorescence lifetime analysis reveals complex decay dynamics in melanin. Both natural and synthetic eumelanin, the most common form of melanin, display heterogeneous, multiexponential decays, which are dominated by an ultrafast component (0.2 to 0.4 ns) alongside slower components extending to 1 ns and in tissues rich in pheomelanin; these lifetimes can occasionally reach 2 to 3 ns.^94^^,^^96^^,^^253^ By contrast, artificial 3,4-dihydroxy-L-phenylalanine melanin (DOPA-melanin) analogs exhibit lifetimes centered near 1.2 ns.^94^^,^^96^^,^^253^ These results indicate that melanin fluorescence arises primarily from a broad, featureless emission tail rather than discrete electronic transitions. Despite its low intensity, melanin consistently provides measurable NIR lifetime contrast in biological imaging, particularly in retinal imaging studies where lifetime variations have been observed in pigmented retinal regions.^258^

Protoporphyrin IX (PpIX), a tetrapyrrolic intermediate in the heme biosynthesis pathway, is an intrinsically fluorescent endogenous molecule. Under physiological conditions, PpIX is present at low concentrations as part of normal cellular metabolism, although elevated endogenous levels have been reported in certain tumor types due to dysregulation of the heme synthesis pathway.^259^^–^^261^ Under violet or blue excitation, PpIX produces characteristic red fluorescence. From a photophysical perspective, reported PpIX fluorescence lifetimes range from ∼3 to 16 ns.^99^^,^^100^^,^^262^^–^^264^ These nanosecond-scale decay dynamics distinguish PpIX from the shorter-lived components that dominate much of the endogenous autofluorescence background and establish it as a distinct source of endogenous lifetime contrast.

Other endogenous molecules, including metabolic cofactors such as NADH and flavins, heme-containing proteins such as hemoglobin and cytochromes, and pigments such as bilirubin and lipofuscin, also contribute to tissue AF backgrounds. However, these species primarily emit in the UV or visible spectral ranges and exhibit weak or negligible intrinsic emission in the NIR. Their fluorescence decay dynamics are often dominated by ultrafast nonradiative relaxation processes or environmental quenching, resulting in limited direct contribution to NIR fluorescence lifetime contrast.^21^^,^^265^^–^^269^ Consequently, although these chromophores can influence time-resolved AF measurements, they generally act as background contributors rather than primary NIR emitters.

Taken together, endogenous fluorophores define the intrinsic AF landscape of biological tissues. Although NIR emissions from these species are generally weak and often arise from spectral tails, their spectral and lifetime characteristics shape the background signals encountered in FLI experiments. Understanding these intrinsic signals therefore provides important context for interpreting lifetime measurements and designing engineered NIR fluorophores capable of achieving robust and specific contrast in biological imaging.

## 4 Applications of NIR Fluorescence Lifetime Imaging

### 4.1 Multiplexing

Collectively, both exogenous and endogenous fluorophores spanning the NIR range, from small organic dyes and fluorescent proteins to inorganic nanomaterials, offer a diverse set of lifetime signatures and photophysical behaviors. These differences in fluorescence lifetime provide a powerful foundation for advanced imaging strategies that move beyond single-color visualization toward multidimensional optical analysis. Building upon these intrinsic lifetime contrasts, multiplexed FLI has emerged as a key imaging strategy for simultaneously resolving multiple molecular species within complex biological systems.

Multiplexed FLI in the NIR window enables simultaneous detection of multiple probes by exploiting complementary spectral and temporal dimensions of contrast. Spectral discrimination is effective when fluorophores have sufficiently separated emission profiles, whereas lifetime provides an orthogonal contrast dimension that can distinguish fluorophores with overlapping spectra when their fluorescence decay characteristics are sufficiently distinct.^270^^–^^273^

Lifetime-based multiplexing has been demonstrated both *in vitro* and in tissue, where fluorophores with overlapping spectral signals can be distinguished through their fluorescence decay characteristics [Fig. 6(a)].^274^ The advantages of lifetime contrast are particularly evident in multiplexed *in vivo* imaging scenarios, where intensity-based methods are fundamentally limited by spectral overlap. A representative example is shown in Fig. 6(b),^29^ where co-administered bone-targeting (OsteoSense, τ∼0.65  ns, $\lambda_{\mathrm{em}}=835\;\mathrm{nm}$^275^) and kidney-targeting (ZW800, τ∼0.5  ns, $\lambda_{\mathrm{em}}=835\;\mathrm{nm}$) probes exhibit overlapping signals in continuous-wave intensity images, making anatomical structures difficult to distinguish. By contrast, fluorescence lifetime decay amplitude maps clearly resolve the distinct spatial distributions of each probe, accurately delineating skeletal structures and renal accumulation. These studies demonstrate the feasibility of NIR lifetime multiplexing under their respective experimental conditions.^29^^,^^274^ However, the separation of a particular probe pair does not establish a general minimum lifetime difference required for successful unmixing. For example, the ∼0.15  ns difference reported between OsteoSense and ZW800 should be interpreted within the acquisition, reference measurements, and analysis used in that study.^29^ Practical discrimination depends on the measured decay signatures and their variability, together with photon counts, background, and the relative signal contributions of the probes.

**Fig. 6:**
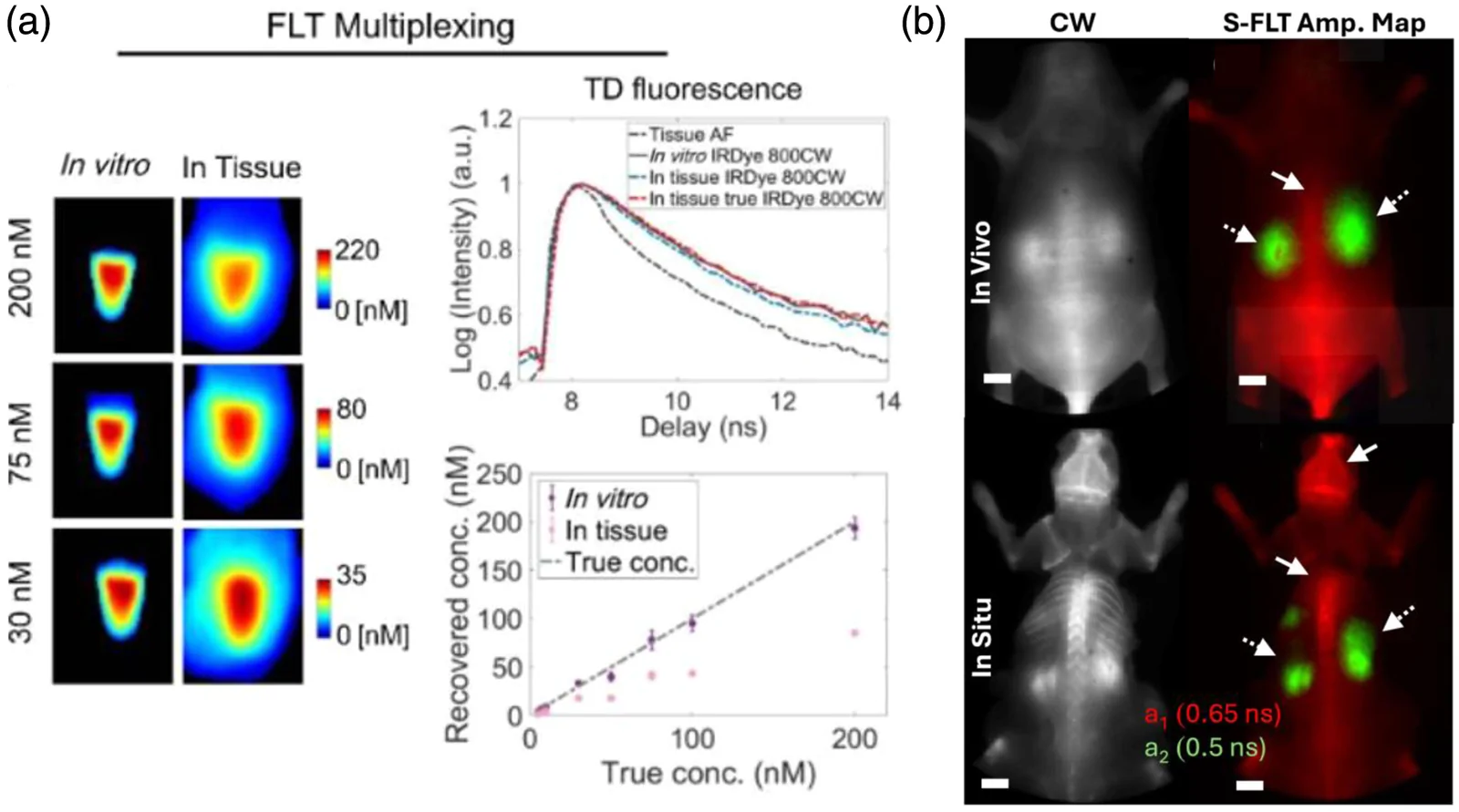
(a) Lifetime-based multiplexing *in vitro* and in tissue. Adapted with permission from Ref. 274, under the Optica Publishing Group Open Access Publishing Agreement. (b) *In vivo* and *in situ* fluorescence intensity maps (left column) and corresponding fluorescence lifetime decay amplitude maps (right column) of a bone-targeting dye OsteoSense (red) and a kidney-targeting dye ZW800 (green) following co-administration. Scale bar=1  cm. Adapted with permission from Ref. 29, under the Optica Publishing Group Open Access Publishing Agreement.

Successful multiplexing should therefore be evaluated through the accuracy and reproducibility of probe assignment in mixed samples. Relevant evidence includes cross-talk between channels, recovery of known signal contributions, and detection of a weak component in the presence of a stronger one. Reference decays should represent the molecular formulations and environments being imaged because conjugation, binding, and aggregation can alter the separation established in solution. For macroscopic imaging, performance should also be assessed across relevant depths and scattering conditions. Reporting these conditions and associated uncertainties would allow readers to distinguish demonstrated multiplexing performance from the potential suggested by tabulated lifetime differences. Where such evaluations are unavailable, conclusions should remain limited to the probe combinations and conditions actually tested.

### 4.2 FRET-Based Molecular Interaction Imaging

FRET enables FLI to detect molecular proximity within ∼2 to 10 nm. Energy transfer from an excited donor to a spectrally compatible acceptor shortens the donor fluorescence lifetime, providing a readout of protein interactions, receptor organization, and ligand- or antibody–target engagement.^5^^,^^276^^–^^278^ Applying FRET to intact animals is challenging because tissue scattering and low photon counts complicate recovery of the donor decay components.^279^^,^^280^ NIR excitation and emission reduce tissue autofluorescence and improve photon penetration. Lifetime-based detection is generally less sensitive to probe concentration, excitation heterogeneity, tissue attenuation, and donor–acceptor-emission cross-talk than intensity-based FRET.^280^^,^^281^ These properties make NIR fluorescence lifetime detection a critical enabling component of macroscopic FLI-FRET (MFLI-FRET) in living animals.^278^^,^^281^^,^^282^

In FLI-FRET, the measured donor decay may contain contributions from donor molecules undergoing FRET and donor molecules not undergoing FRET.^278^^,^^279^ A bi-exponential decay model can estimate the relative amplitude of the short-lifetime component, termed the FRET donor fraction ($F_{D}$), as well as FRET efficiency, which is calculated as in Eq. (4).

$$E=1-\frac{\tau_{\mathrm{DA}}}{\tau_{D}},$$ (4)

where $\tau_{D}$ is the unquenched donor lifetime and $\tau_{\mathrm{DA}}$ is the FRET-shortened lifetime.^208^^,^^278^^–^^280^^,^^283^ These parameters provide complementary information: $F_{D}$ is an amplitude-derived estimate of the detected donor population undergoing FRET, whereas E describes the lifetime reduction of the FRETing donor population and is sensitive to donor–acceptor distance and orientation. A donor-only reference should be established for each probe formulation under relevant labeling and environmental conditions and remeasured whenever those conditions materially change.^280^^,^^284^ Interpretation also requires consideration of the donor-to-acceptor ratio, degree and site of labeling, probe affinity, epitope geometry, receptor organization, and target accessibility.^27^^,^^69^

NIR FLIM-FRET microscopic measurements in cells can establish the donor-only lifetime, optimize labeling and donor-to-acceptor ratios, test assay specificity, and relate the short lifetime component to receptor-associated interactions and cellular distribution.^284^^,^^285^ Macroscopic MFLI-FRET then uses these characterized probe pairs and lifetime parameters for wide-field imaging of intact tumors and living animals.^208^^,^^278^^,^^285^ Thus, microscopic FLIM-FRET provides biological and photophysical validation for the macroscopic measurement, whereas MFLI-FRET enables noninvasive, longitudinal mapping of the validated interaction across intact tumors and mice.^3^^,^^285^

Initial NIR MFLI-FRET studies used donor- and acceptor-labeled transferrin to measure transferrin receptor engagement in tumor xenografts.^69^^,^^208^^,^^278^ Subsequent studies used AF700/AF750-labeled trastuzumab pairs to quantify HER2 engagement in HER2-positive cells and intact tumors.^284^^,^^285^ In these assays, donor intensity primarily reflects probe delivery and accumulation, whereas $F_{D}$ identifies the fraction of donor-labeled probes undergoing receptor-associated FRET.^280^^,^^284^ The nonfluorescent acceptor IRDye QC-1 reduced acceptor-emission cross-talk and enabled multiplexed imaging of tumor glucose metabolism and receptor–ligand engagement.^286^ Combined microscopic FLIM-FRET and whole-animal MFLI-FRET further demonstrated reduced trastuzumab–HER2 engagement in poorly vascularized, collagen-rich tumors despite comparable HER2 expression, with the macroscopic findings independently supported by *ex vivo* immunohistochemistry.^285^ These studies demonstrated that validated NIR FLI-FRET assays can distinguish receptor-associated engagement from total probe accumulation *in vivo*.

Quantitative MFLI-FRET is challenged by low photon counts, tissue-dependent temporal dispersion, spatially variable background, photon migration, and probe populations occupying different biological states.^279^^,^^280^ These factors can bias lifetime components and $F_{D}$ if the instrument response, reference lifetime, photon propagation, and decay model are not appropriately characterized. High-spatial-frequency structured illumination and subdiffusive reconstruction methods are being developed to suppress superficial fluorescence and improve depth specificity in MFLI.^15^

### 4.3 Microenvironmental Sensing

Beyond distinguishing fluorophores and detecting molecular interactions, NIR FLI can exploit environment-dependent changes in fluorescence decay to report local biochemical and biophysical conditions. These applications rely on fluorophores with excited-state decay that responds to defined environmental variables or to changes in molecular binding and the cellular microenvironment.

A direct example of lifetime-based environmental sensing is provided by a peptide-functionalized CdSe/ZnS quantum dot (CdSe/ZnS-PH), in which protonation of the D-penicillamine–histidine surface ligand produces a reversible decrease in fluorescence lifetime upon acidification [Fig. 7(a)].^287^ In C3H10T1/2 cells, the probe exhibited a linear lifetime response between pH 6.4 and 7.2 with a sensitivity of ∼2.0  ns per pH unit, enabling an intracellular pH of 6.97±0.14 to be determined. Its comparatively long cellular lifetime (10.84±0.30  ns) remained stable for 24 h, supporting longitudinal intracellular measurements with limited interference from short-lived cellular AF.^287^

**Fig. 7:**
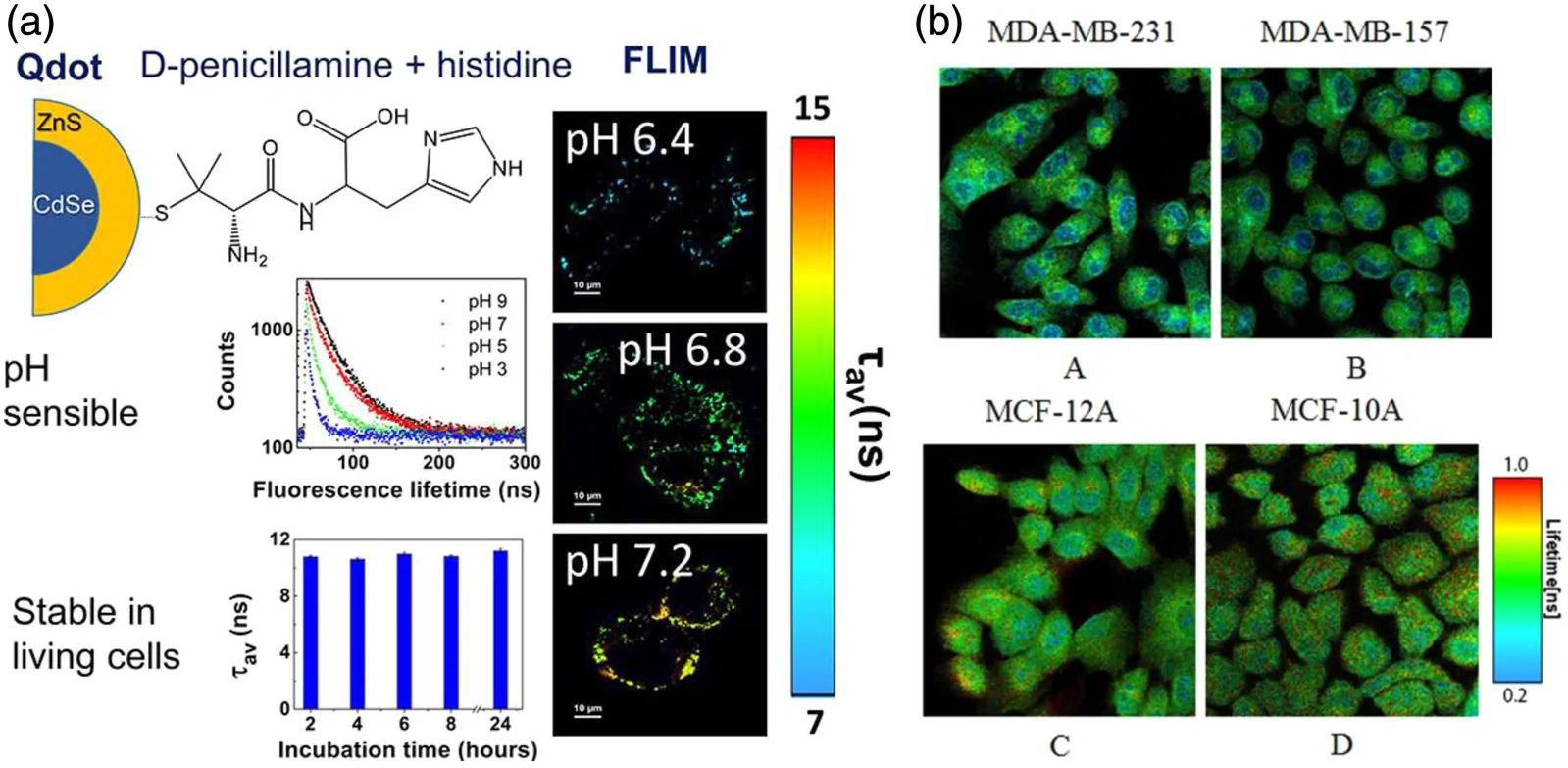
(a) CdSe/ZnS quantum dot-based pH probe designed for long-term FLIM in C3H10T1/2 cells at intracellular pH values of 6.4, 6.8, and 7.2. Adapted with permission from Ref. 287, licensed under CC BY 4.0. (b) Representative MB fluorescence lifetime images of the breast cancer cell lines (A) MDA-MB-231 and (B) MDA-MB-157 and the normal breast epithelial cell lines (C) MCF-12A and (D) MCF-10A. MB fluorescence lifetimes are shorter in the cancer cells than in the normal epithelial cells. Adapted with permission from Ref. 288, licensed under CC BY 4.0.

Methylene blue (MB) illustrates how fluorescence lifetime can reflect several environmental influences. Measurements in homogeneous media and DNA-bound complexes demonstrate sensitivity to solvent properties and molecular binding.^88^^,^^94^ In cellular FLIM, the longer decay component was ∼0.75  ns in the investigated breast cancer cells, compared with ∼0.89 to 0.95 ns in normal epithelial cells, shown in Fig. 7(b).^288^ These differences provide contrast between the studied cellular populations. However, because the measurements do not isolate individual physicochemical variables, they cannot uniquely attribute the observed contrast to polarity, viscosity, or binding. MB lifetime should therefore be interpreted as a composite readout of the cellular environment under these conditions.

The quantum-dot and MB studies illustrate different evidential requirements. The calibrated lifetime–pH relationship of CdSe/ZnS-PH supports quantitative pH estimation within the tested range and conditions,^287^ whereas the MB results support discrimination between biological states without establishing a unique physicochemical cause. The quantum-dot probe emits near 627 nm and therefore provides a visible-emission methodological example relevant to NIR sensor development, rather than a direct demonstration within the NIR range defined here. Quantitative NIR sensors similarly require calibrated responses, characterized uncertainty, and assessment of competing influences. Probes intended for biological-state discrimination instead require reproducible contrast across relevant specimens and controls, even when the molecular origin of that contrast remains incompletely resolved.

### 4.4 Clinical Translation

The microenvironment-sensitive lifetime behavior described above becomes clinically relevant when it enables pathological tissue to be distinguished from surrounding nonpathological tissue. Current translational evidence encompasses both lifetime-resolved tissue characterization and established fluorescence-guided surgical applications that continue to rely primarily on fluorescence intensity. Clinically approved fluorophores and targeted fluorescent agents define the translational benchmark for NIR imaging, and representative applications in colorectal, hepatopancreatobiliary, and endocrine surgery are shown in Fig. 8(a).^32^

**Fig. 8:**
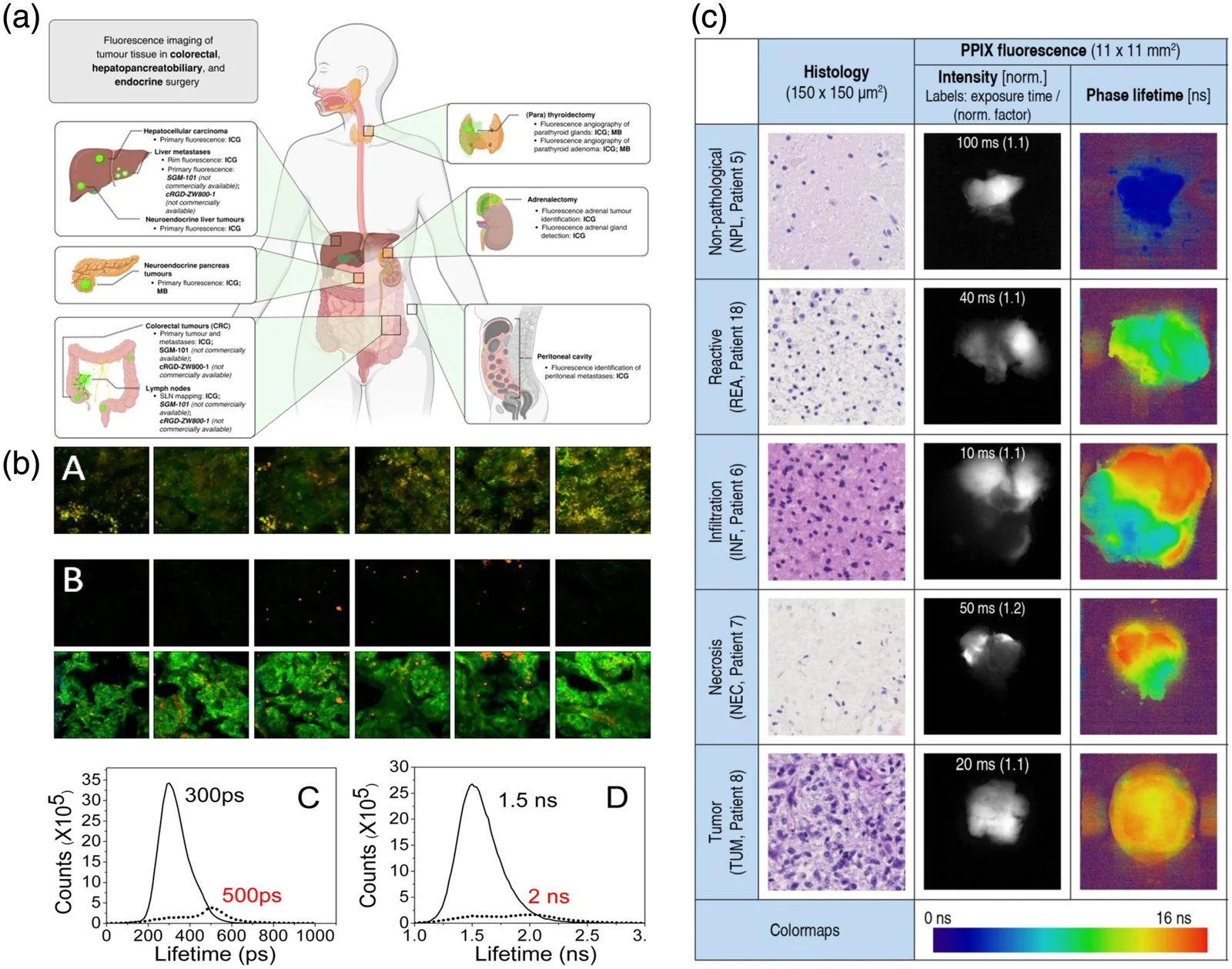
(a) Overview of clinical applications of fluorescence-guided surgery in colorectal, hepatopancreatobiliary, and endocrine procedures using clinically approved fluorophores. Adapted with permission from Ref. 32, licensed under CC BY-NC 4.0. (b) Endogenous bilirubin fluorescence lifetime contrast in cryosectioned liver tissue reveals altered short lifetime component $\tau_{1}$ distributions between nontumorous regions (A) and poorly differentiated hepatocellular carcinoma (B). Corresponding short $\tau_{1}$ (C) and long $\tau_{2}$ (D) lifetime distributions in tumor tissue. Adapted with permission from Ref. 108. (c) PpIX fluorescence intensity and phasor lifetime maps of brain tumor tissue with corresponding IHC. Adapted with permission from Ref. 31, licensed under CC BY 4.0; only the PpIX and histology panels are cropped and used with attribution.

#### 4.4.1 Lifetime-resolved tumor imaging

Endogenous bilirubin provides an example of lifetime-based pathological tissue characterization near the lower boundary of the NIR spectral range. Under 1230-nm two-photon excitation, bilirubin dimers produce red fluorescence around 660 nm.^108^ Time-resolved measurements in cryosectioned liver tissue revealed altered bilirubin lifetime distributions between poorly differentiated hepatocellular carcinoma (HCC) and corresponding nontumorous regions [Fig. 8(b)].^108^ The short-lifetime component increased from ∼0.3  ns in nontumorous tissue to ∼0.5  ns in hepatocellular carcinoma, indicating sensitivity to disease-associated changes in bilirubin aggregation, molecular binding, and the local tissue microenvironment.

PpIX-associated fluorescence has been used for the optical detection of epithelial lesions and early cancer diagnostics.^289^^,^^290^ However, the fluorescence signal from naturally occurring PpIX is generally weak and has been enhanced in many clinical imaging applications through administration of 5-aminolevulinic acid (5-ALA), a metabolic precursor that increases intracellular porphyrin production and leads to preferential accumulation of PpIX in tumor tissue.^259^^–^^261^^,^^291^ Under violet or blue excitation, accumulated PpIX produces a characteristic red emission that enables direct visualization of malignant tissue. A randomized multicenter phase III trial in patients with malignant glioma demonstrated that 5-ALA fluorescence-guided surgery improved complete resection rates and 6-month progression-free survival compared with conventional white-light surgery. Six-month progression-free survival was 41.0% versus 21.1%, an absolute difference of 19.9 percentage points.^292^

Beyond this established intensity-based use, the nanosecond-scale decay dynamics of PpIX enable lifetime-based contrast mechanisms that complement conventional intensity imaging and motivate FLI approaches for improved discrimination between malignant and nonpathological tissue.^31^^,^^99^^,^^293^^,^^294^ As shown in Fig. 8(c),^31^ malignant regions exhibit prolonged PpIX fluorescence lifetimes compared with nonpathological tissue, providing improved contrast for residual tumor beyond conventional intensity imaging.

Indocyanine Green (ICG), approved by the FDA in 1959, remains the most widely used NIR dye for perfusion assessment and FGS.^30^^,^^274^^,^^295^^–^^302^ A study of patient-derived surgical specimens obtained after systemic ICG administration demonstrated fluorescence lifetime contrast between malignant and noncancerous tissues across multiple solid tumor types. Tumor-associated ICG lifetimes were longer than those in corresponding noncancerous tissues, with histopathological comparison supporting a reported classification accuracy exceeding 97%. These findings demonstrate lifetime-based tumor discrimination in excised human tissue and support further development of ICG lifetime imaging for surgical guidance. Prospective intraoperative studies are required to establish whether this contrast improves margin assessment and surgical outcomes.^30^

#### 4.4.2 Intensity-based fluorescence-guided surgery

Established intensity-based fluorescence-guided surgery provides a clinical context for translating NIR lifetime imaging. Endogenous parathyroid autofluorescence enables tissue identification without an administered probe, whereas ICG-based imaging illustrates how surgical contrast depends on biodistribution, clearance, and imaging time.^110^^–^^112^^,^^252^^–^^256^ Targeted and activatable agents further demonstrate strategies for localizing fluorescence to diseased tissue.^262^^–^^274^^,^^278^^–^^280^ These applications establish practical requirements for probe delivery and surgical visualization, but their clinical performance does not establish the additional diagnostic value of fluorescence lifetime.

Available lifetime measurements nevertheless identify opportunities for further investigation. IRDye 800CW has reported lifetimes of ∼0.435  ns in PBS and 1.1 ns in DMSO,^16^^,^^87^ whereas a lifetime of 3.56 ns has been reported for panitumumab-conjugated IRDye 700DX.^84^^–^^86^ LS301 has reported lifetimes of 0.21 ns in water and 0.84 ns in glioma cells,^282^^,^^283^ and ZW800-1C has shown lifetime differences among labeled neuropathological structures.^93^ These measurements provide formulation- and environment-specific observations; they should not be treated as interchangeable intrinsic dye constants or evidence of validated clinical lifetime discrimination.

The distinction between probe characterization and application-mimicking measurements is particularly important for Cytalux. A lifetime of ∼1.95  ns has been reported in OTL-38–mimicking lung solid phantoms.^90^ This value characterizes the reported phantom measurement and should not be assigned to Cytalux itself without confirmation of the emitting species and formulation. Similarly, the targeting or activation mechanisms of other surgical probes do not establish their suitability for lifetime imaging when their decay characteristics remain uncharacterized.

The translational priority is therefore to determine whether lifetime measurements provide reproducible information beyond fluorescence intensity under clinically relevant conditions. This requires characterization of the administered formulation, validation against the relevant biological or pathological endpoint, and direct assessment of the added value of lifetime contrast.

## 5 Imaging Instrumentation and Fluorescence Lifetime Analysis

### 5.1 Acquisition Modalities for NIR FLI

Fluorescence lifetime measurements are commonly implemented using either time-domain or frequency-domain acquisition approaches. Time-domain methods sample the temporal fluorescence response following pulsed excitation, with the measured fluorescence intensity plotted as a function of time, as shown in Fig. 9(a). Frequency-domain methods measure the phase delay and modulation response of fluorescence under periodically modulated excitation, which can be represented in phasor space, as shown in Fig. 9(b).^2^^,^^59^

**Fig. 9:**
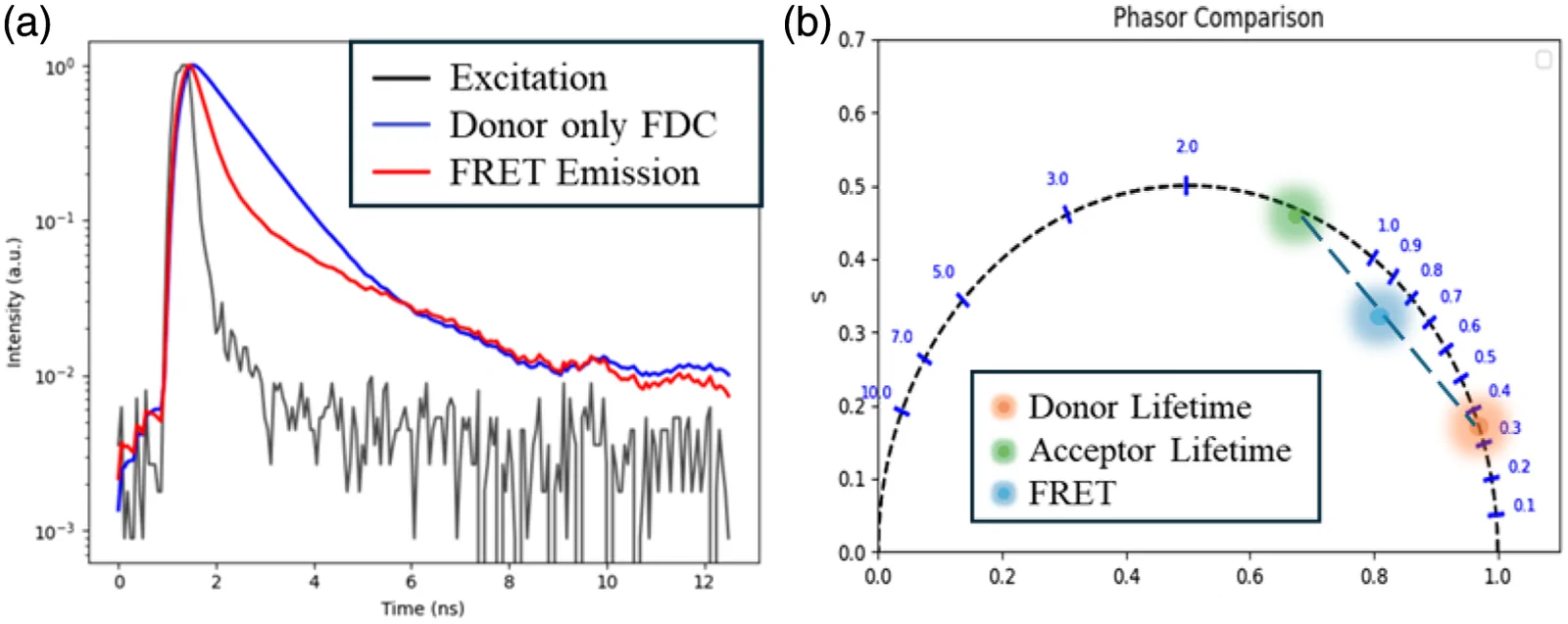
Representative outputs of time- and frequency-domain fluorescence lifetime measurements. (a) Excitation response and representative fluorescence decay curves displayed as a function of time. (b) Representative frequency-domain phasor plot illustrating the distribution of fluorescence lifetime signals according to their phase and modulation responses.

The two principal time-domain approaches are time-correlated single-photon counting (TCSPC) and time-gated acquisitions. TCSPC records the arrival time of each detected fluorescence photon relative to the excitation pulse. Photon-arrival events accumulated over repeated excitation cycles form a temporal histogram representing the fluorescence response.^270^^,^^303^^,^^304^ Because only individual photon events are recorded, the count rate must be controlled to limit pile-up, which occurs when multiple photons arrive during one excitation cycle but only the first event is registered.^305^ The excitation period must also be sufficiently long to avoid substantial overlap between signals generated by consecutive excitation pulses.

Time-gated acquisition instead integrates fluorescence photons within a series of temporal windows synchronized with the excitation pulse. Each gate samples the fluorescence signal over a defined duration and delay, and sequentially shifting the gate produces a set of temporally resolved measurements across the fluorescence response.^306^^–^^309^ The gate width, temporal spacing, and number of acquired gates determine the temporal sampling and photon collection. Narrow and closely spaced gates provide more detailed temporal information, whereas broader or fewer gates increase photon collection and acquisition speed.

Frequency-domain acquisition uses periodically modulated excitation rather than directly sampling the temporal fluorescence response. The emitted fluorescence follows the excitation modulation but exhibits a phase delay and reduced modulation depth because of the finite excited-state duration.^310^^,^^311^ These measurements may be performed at one modulation frequency or across multiple frequencies to characterize the frequency response more completely.

Accordingly, TCSPC provides densely sampled photon-arrival distributions but can require longer acquisition times and careful control of count rate. Time-gated acquisition enables parallel temporal sampling across an imaging field, with its temporal information determined by the gate configuration. Frequency-domain acquisition records phase and modulation information without directly acquiring the full temporal decay. Selection among these approaches therefore depends on the expected lifetime range, available photon counts, required acquisition speed, and imaging field of view.

### 5.2 Detector Technologies and Imaging Architectures

The implementation of NIR FLI depends on detector characteristics including NIR sensitivity, photon-detection efficiency, temporal response, dark-count rate, active area, and compatibility with point-scanning or widefield acquisition. These characteristics are particularly important for resolving the weak emission and sub-nanosecond lifetimes commonly encountered with NIR fluorophores. Detector technologies are considered here according to their physical architecture rather than acquisition domain because the same detector class may support different lifetime-measurement schemes when combined with appropriate timing, gating, or modulation electronics.

Photomultiplier tubes (PMTs) and hybrid photodetectors are established single-photon detectors for TCSPC-based measurements and are commonly implemented in point-scanning or multichannel imaging systems, for example, a TCSPC FLIM system with 16 spectral channels shown in Fig. 10(a).^270^ Their temporal resolution is governed primarily by the transit-time spread and associated timing electronics, whereas their spectral sensitivity is determined by the photocathode material.^315^^,^^316^

**Fig. 10:**
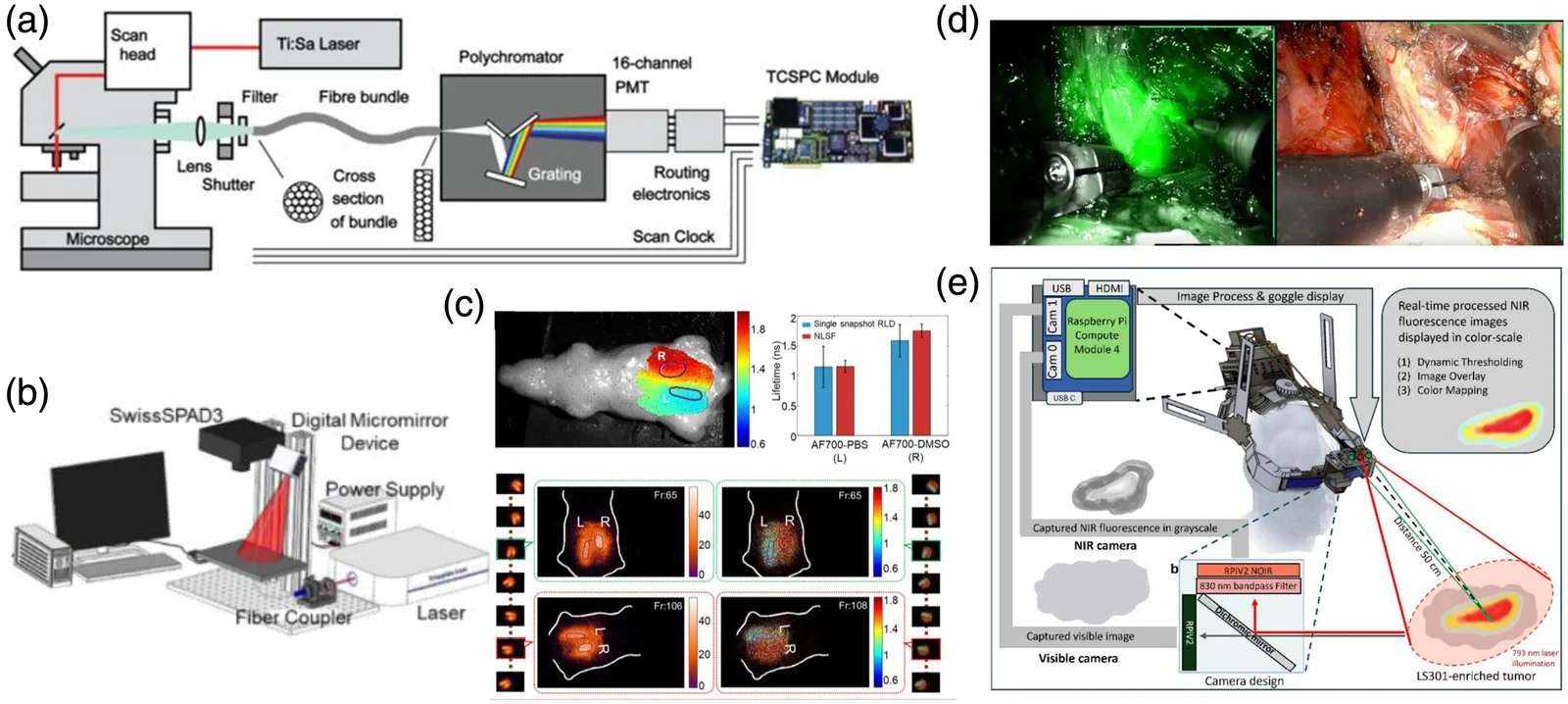
(a) Sixteen wavelength channel FLIM setup. Reproduced with permission from Ref. 270. (b) Single-snapshot macroscopic FLI using a SwissSPAD3 (SS3) camera in a widefield setup. (c) A tissue-mimicking mouse-shaped phantom containing two embedded tubes filled with AF700 in PBS and DMSO was imaged to demonstrate macroscopic lifetime mapping under dynamic motion conditions through imaging system in panel (b). Both panels (b) and (c) are adapted with permission from Ref. 312, licensed under CC BY 4.0. (d) ICG fluorescence imaging during transoral robotic surgery for localization of retropharyngeal nodal metastasis from papillary thyroid carcinoma. White-light view and near-infrared fluorescence view using the FIREFLY imaging mode of the Da Vinci SP system. Adapted with permission from Ref. 313, licensed under CC BY 4.0. (e) Schematic illustration of the cancer vision goggles imaging system and data acquisition workflow for quantitative near-infrared fluorescence-guided surgery. The diagram depicts the wearable imaging platform and associated data acquisition pipeline used for real-time fluorescence visualization during surgical procedures. Adapted with permission from Ref. 314, licensed under CC BY 4.0; selected components of the original figure are used with attribution.

For widefield time-gated imaging, ICCD cameras combine an image intensifier with a CCD or related camera sensor. The intensifier contains a photocathode, microchannel plate, and phosphor screen, and rapid voltage switching at the photocathode enables sub-nanosecond optical gating.^306^^,^^307^^,^^309^^,^^317^ ICCDs provide high optical gain and a large continuous imaging area but require vacuum-tube components and can be affected by microchannel-plate gain variation, phosphor persistence, and camera readout noise.^318^^,^^319^

More recent systems implement time gating directly within solid-state CMOS image sensors, avoiding the external image intensifier used by ICCD cameras. One example is the tauCAM system, which employs a water-cooled 128×128 current-assisted photonic sampler (CAPS) pixel array designed for macroscopic FLI. The CAPS sensor is fabricated in a commercial 350-nm CMOS process, and current assistance enables rapid transport and temporal gating of photogenerated carriers within the substrate. Its sub-nanosecond gating and NIR quantum efficiency above 40% support measurements of short NIR lifetimes while multiple-exposure imaging with nondestructive readout extends the available dynamic range to over 100 dB.^320^^,^^321^

Alongside CAPS-based cameras, SPAD arrays represent a second major CMOS-compatible architecture for time-resolved imaging. SPAD arrays are solid-state detectors that offer imaging capabilities at the level of individual photons, with unparalleled photon-counting and time-resolved performance.^322^^–^^325^ Recently, significant progress has been made in fabrication processes, enabling the integration of individual SPAD pixels into arrays large enough to function as full imaging sensors. However, due to the complex electronics and large data streams associated with TCSPC data acquisition, the primary technique for single-detector systems, TCSPC-based SPAD cameras have typically been limited to small formats.^305^^,^^326^^,^^327^ By contrast, time-gated SPAD arrays are easier to scale to larger fields of view and provide significantly higher global photon count-rate capabilities. Recent commercial systems, such as SPAD512S (or $\mathrm{SPAD}512^{2}$), enable FLI with relatively high pixel density (512×512  pixels), single-photon sensitivity, and readout speeds of up to 100,000 1-bit frames per second. Despite relatively wide gate widths, such SPAD arrays have been demonstrated to enable quantitative FLI of short lifetimes.^16^^,^^328^^,^^329^

CMOS fabrication enables SPAD arrays to integrate photon-detection and processing electronics, such as avalanche quenching and recharge circuits, temporal gating or photon-timing circuitry, and local counters or memory, on the same chip as the detector array. This integration supports parallel photon detection and high-rate imaging, but the additional circuitry competes with the photosensitive area and increases readout complexity, producing tradeoffs among pixel size, fill factor, timing capability, and data throughput.^330^ Dark counts, afterpulsing, crosstalk, dead time, and pixel-to-pixel response variation remain important performance considerations.^330^^–^^332^ A representative implementation is the SwissSPAD3 (SS3) camera, which employs a large-format time-gated SPAD array with a dual-gate acquisition architecture.^312^ The system simultaneously records fluorescence within a user-defined temporal gate (G2) and over the full laser-repetition period (INT), providing temporally correlated gated and intensity measurements within a single acquisition. When integrated into a widefield macroscopic FLI system, SS3 supports single-snapshot acquisition across a large field of view, as illustrated in Fig. 10(b).^312^

Another emerging detector technology with strong potential for time-resolved fluorescence imaging is the superconducting nanowire single-photon detector (SNSPD). SNSPDs consist of superconducting nanowires operated below their critical temperature and biased near the superconducting transition, such that absorption of a single photon generates a measurable electrical pulse.^333^^–^^335^ Owing to their high single-photon sensitivity, low dark count rates, afterpulse-free operation, and exceptionally low timing jitter, SNSPDs are particularly attractive for low-light and high-speed fluorescence measurements, especially in the near-infrared where fluorophores often exhibit weak emission and short excited-state lifetimes.^336^^,^^337^ Although their application in FLI remains limited, SNSPDs have begun to be explored in fluorescence-based measurements such as time-resolved photoluminescence, single-molecule detection, fluorescence correlation spectroscopy, and deep-tissue SWIR microscopy, where their improved temporal precision and signal-to-noise performance can offer clear advantages over conventional NIR photomultiplier-based detectors.^337^^–^^339^ However, despite these promising characteristics, widespread integration of SNSPDs into practical FLI platforms remains challenging. Their operation requires cryogenic cooling and specialized readout electronics, whereas efficient photon collection is often constrained by small active areas, limited fill factors, optical coupling losses, polarization-dependent detection efficiency, and detector dead time.^337^^,^^338^ In addition, current SNSPD implementations are often highly customized research systems rather than broadly deployable imaging solutions, and scaling these detectors into large-area or high-pixel-count array formats suitable for widefield, endoscopic, or clinically integrated fluorescence imaging remains a significant technical challenge.^337^^,^^338^

Collectively, these detector technologies support architectures ranging from point-scanning microscopic FLIM to widefield and macroscopic NIR FLI. PMT and hybrid-detector systems remain widely used for scanning or multichannel TCSPC, whereas ICCD, CAPS, and SPAD cameras enable parallel acquisition across larger imaging fields.

To date, emerging FGS systems have focused primarily on real-time intensity-based NIR visualization. Intensity-based surgical platforms also illustrate practical requirements for clinical FLI integration. The FIREFLY system,^313^ shown in Fig. 10(d), demonstrates fluorescence visualization within a robotic surgical workflow, whereas cancer vision goggles,^314^ shown in Fig. 10(e), illustrate hands-free display and control of acquisition geometry.^302^^,^^340^^,^^341^ The cited implementations provide intensity-based guidance; their relevance to FLI lies in these integration features. Extending such platforms to lifetime imaging would additionally require compatible excitation and timing hardware, instrument-response calibration, and sufficient photon collection to achieve useful lifetime precision within the available acquisition time. Clinical usability should therefore be assessed through the acquisition time and reliability of complete lifetime maps, alongside spatial resolution, field of view, and compatibility with surgical workflow.

### 5.3 Fluorescence Lifetime Estimation and Computational Analysis

Following time-resolved acquisition, fluorescence lifetime analysis converts the measured temporal or frequency-domain signal into quantitative lifetime parameters. For time-domain measurements, the detected fluorescence decay at each pixel can be represented as

$$I_{\mathrm{meas}}(t)=\mathrm{IRF}(t)⊗[\overset{N}{\underset{i=1}{\sum }}A_{i}e^{-t/\tau_{i}}]+B(t),$$ (5)

where $A_{i}$ and $\tau_{i}$ are the pre-exponential amplitude and fluorescence lifetime of the i’th component, respectively, B(t) represents the background contribution, and ⊗ denotes convolution with the instrument response function (IRF).

Equation (5) assumes linear excitation and detection, with temporal dispersion caused by photon propagation within the sample either negligible or independently corrected. Here, the IRF accounts for instrumental broadening, including the excitation pulse and detection timing response. In macroscopic tissue imaging, excitation and emission photon migration can introduce additional delays and broadening that depend on tissue optical properties, fluorophore depth, and measurement geometry. Reconvolution with an instrument-only IRF does not account for these contributions. When propagation effects materially influence the analyzed decay, lifetime recovery requires a model incorporating photon transport or a correction validated under representative measurement conditions. Without such validation, the recovered parameters should be reported as apparent lifetimes, acknowledging the potential contribution of photon migration.

Monoexponential models are generally sufficient for fluorophores in homogeneous environments, whereas multiexponential models may be required when multiple fluorophores, conformational states, binding environments, or AF components contribute to the measured decay.^59^^,^^342^^,^^343^

Decay parameters are commonly estimated using nonlinear least-squares fitting or maximum-likelihood estimation. Reconvolution with the measured IRF is particularly important for NIR fluorophores with sub-nanosecond lifetimes that are comparable to the temporal width of the instrument response. Pixel-wise fitting provides spatial lifetime maps, whereas global fitting can constrain selected lifetime components across pixels, spectral channels, or repeated measurements to reduce parameter degeneracy in heterogeneous data.^342^ Depending on the decay model, the reported output may include individual lifetime components and amplitudes, an amplitude-weighted mean lifetime, or the fractional contribution of each component.

For frequency-domain measurements, lifetime is calculated from the phase delay ϕ and demodulation factor M of the fluorescence relative to the modulated excitation. For an ideal monoexponential decay, the corresponding phase and modulation lifetimes are shown in Eqs. (6) and (7), where ω is the angular modulation frequency. Agreement between these quantities is expected for a monoexponential decay, whereas their divergence can indicate heterogeneous or multicomponent fluorescence.^59^^,^^310^

$$\tau_{\phi }=\frac{\tan(\phi )}{\omega },$$ (6)

$$\tau_{M}=\sqrt{M^{-2}-1}/\omega .$$ (7)

Recent efforts to reduce the acquisition and processing time of time-gated FLI have explored rapid lifetime determination (RLD), which estimates an apparent lifetime from fluorescence signals acquired over a small number of temporal gates rather than through iterative decay fitting. When combined with sparse- or simultaneous-gate acquisition, RLD can support rapid or single-snapshot lifetime imaging, as demonstrated by the dynamic phantom measurements shown in Fig. 10(c). RLD generally assumes monoexponential decay behavior and remains sensitive to background subtraction and the timing and width of the selected gates.^312^

Phasor-based lifetime and AF unmixing offers an orthogonal approach that separates fluorophores according to their nanosecond-scale decay kinetics, enabling accurate distinction even when emission spectra overlap.^271^^,^^344^ In phasor analysis, each fluorescence decay is transformed into normalized sine and cosine coordinates in a two-dimensional phasor space shown in Fig. 11(a).^345^ Ideal single-exponential decays lie on the universal semicircle, whereas mixtures of lifetime components occupy positions within the semicircle according to their relative contributions. This graphical representation enables rapid identification and separation of lifetime populations without explicitly fitting a predefined multiexponential decay model.^342^^,^^346^

**Fig. 11:**
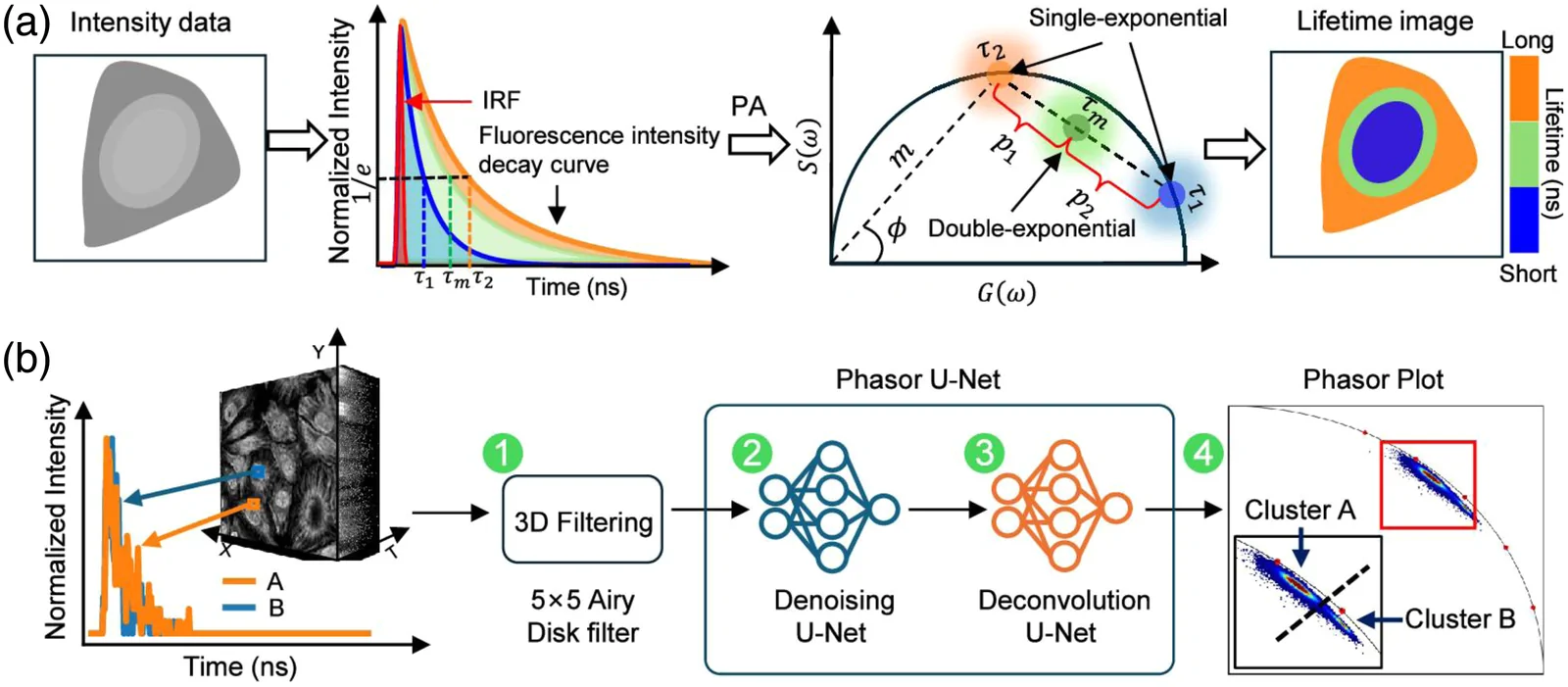
(a) Traditional phasor analysis and (b) phasor-based U-net unmixing of TCSPC-FLIM imaging data. Reproduced with permission from Ref. 345.

Beyond direct lifetime estimation, computational unmixing methods combine temporal and spectral information to separate fluorophores with partially overlapping signatures. Recently, deep-learning strategies have further enhanced multiplexing performance by simultaneously analyzing spectral and lifetime information to achieve high-throughput signal separation with improved noise tolerance, as illustrated by the phasor-based U-Net shown in Fig. 11(b).^345^^,^^347^^–^^349^ Related learning-based approaches can estimate lifetime parameters directly from sparsely sampled time-gated measurements and incorporate spatially varying IRFs to improve reconstruction across nonplanar samples. However, their performance depends on the representativeness of the training and validation data, particularly across different fluorophores, photon levels, and imaging systems.

Although these analytical approaches enable increasingly rapid and flexible lifetime estimation, their reliability remains dependent on photon statistics, instrument characterization, model assumptions, and the method used to evaluate uncertainty. These limitations and the need for standardized reporting across NIR FLI platforms are discussed in the following section.

Method selection should follow the quantity that the experiment aims to recover. For rapid mapping of an apparent lifetime, sparse time-gated acquisition combined with rapid lifetime determination can reduce acquisition and processing demands when a single-exponential approximation adequately describes the signal. Its suitability depends on gate placement, background correction, and the precision achieved within the available photon budget. Performance should therefore be expressed as the time required to produce a complete lifetime map at a stated precision and spatial resolution, rather than detector readout speed alone. A rapid apparent-lifetime estimate should not be interpreted as resolving individual decay components.

Decay-component estimation and FRET quantification require sufficient temporal information and detected photons to constrain the chosen model. TCSPC, adequately sampled time-gated measurements, and multifrequency frequency-domain acquisition can provide complementary approaches, with performance determined by instrument-response characterization, sampling, and parameter identifiability. For FRET, a matched donor-only reference and appropriate controls are additionally required to distinguish transfer-induced shortening from other photophysical changes. Comparisons should report bias and precision for the relevant lifetimes and component contributions, together with photon counts, acquisition time, and imposed fitting constraints. Recovering an average lifetime accurately does not establish equivalent accuracy for multiple component lifetimes or FRET fractions.

For probe unmixing, phasor and combined spectral–temporal approaches are useful when reference signatures remain distinguishable under the measurement conditions. Their practical performance is determined by cross-talk, recovery of known signal contributions, and detection of weak components alongside stronger signals. Phasor analysis avoids explicit multiexponential fitting, but molecular assignment still depends on suitable references and control of instrument- and tissue-dependent distortions. Learning-based methods likewise require validation across relevant photon levels, probe mixtures, and instrument responses. Where methods have not been compared under matched conditions, their reported performance should be interpreted within the demonstrated measurement range. This application-specific assessment provides a more useful basis for selecting an imaging strategy than nominal timing resolution, pixel count, or frame rate considered individually. The newly developed platform pyFLI^350^ addresses this need with a unified interface for FLI data analysis, simulation, and visualization, standardizing data handling across acquisition systems and supporting both analytical and deep-learning-based fitting.

## 6 Current Challenges to Quantitative and Reproducible NIR FLI

FLI has established itself as a robust quantitative modality, and its extension into the NIR region has experienced rapid growth over the last decade, with expanding applications across molecular imaging, fluorescence-guided surgery, biosensing, and preclinical *in vivo* imaging. This progress reflects increasing recognition of fluorescence lifetime as a concentration-independent and environmentally sensitive source of contrast, particularly in biological settings where intensity-based measurements are often confounded by scattering, absorption, and fluorophore concentration. However, despite this momentum, the continued development and broader adoption of NIR FLI remain constrained by several fundamental scientific and technological challenges.

The foremost limitation is the absence of standardized and reproducible lifetime data for NIR fluorophores. Reported lifetimes for widely used agents such as ICG or IRDye 800CW vary across buffers, binding states, and experimental conditions, as expected for environmentally sensitive fluorophores. However, the larger differences reported across studies often reflect incomplete reporting and inconsistent lifetime analysis. Such inconsistencies hinder inter-laboratory comparison and obscure physiological interpretation.

Cross-study comparison is further complicated when reports do not fully specify the excitation and detection wavelength ranges, detector characteristics, detected signal and background levels, and acquisition parameters. The excitation and detection bands define which absorbing and emitting species contribute to the measured decay, whereas the detector response and acquisition settings determine the temporal resolution and statistical precision of the measurement.^14^ These considerations are particularly important for sub-nanosecond NIR decays, for which limited temporal resolution or inaccurate IRF characterization can bias the estimated lifetime, whereas low signal relative to background reduces measurement precision.^351^ Assessment of repeatability also requires the number of independent measurements and the variability observed across repeated measurements.

At the fluorophore level, the energy-gap-law penalty links NIR emission to increased nonradiative relaxation, resulting in the lower quantum yields and shorter fluorescence lifetimes characteristic of many NIR organic dyes.^127^ These properties impose stringent requirements on time-resolved acquisition. When the fluorescence decay approaches the temporal width of the IRF, errors in IRF measurement or alignment directly bias the recovered lifetime. Because the IRF may vary with detection wavelength, detector channel, and position across the field of view, applying a single global IRF can introduce wavelength- or pixel-dependent bias, particularly for sub-nanosecond decays. Reduced detector sensitivity at longer wavelengths and photon losses caused by tissue absorption and scattering further decrease photon counts, increasing estimator variance and limiting the reliable separation of fluorophores with similar lifetimes.^205^ Cross-platform comparison is further complicated by inconsistent calibration practices and the environmental sensitivity of fluorophores used as lifetime references.

Acquisition-dependent artifacts introduce additional systematic errors. In TCSPC measurements, pile-up occurs when the photon-detection rate is too high relative to the excitation repetition rate. This artifact preferentially records photons arriving early in the excitation cycle and consequently shortens the recovered lifetime. Detector dead time, timing jitter, dark counts, and background photons further distort the measured temporal histogram. In time-gated systems, gate width, gate placement, temporal step size, and gate-to-gate sensitivity variations determine the accuracy with which short decay components are sampled.^304^^,^^315^^,^^319^^,^^352^ In detector channels that use leading-edge threshold discrimination, time walk occurs when pulses with different amplitudes or rise times cross the timing threshold at different times despite corresponding to the same photon-arrival time. This amplitude-dependent electronic delay changes the position and width of the detector timing response and biases the estimation of short fluorescence lifetimes when it differs between the IRF and fluorescence measurements.^353^^,^^354^

Biological heterogeneity presents a separate analytical challenge. When multiple fluorophores, binding states, conformations, or AF sources contribute to the signal within a single pixel, the measured decay contains multiple lifetime components that cannot be resolved by a single-exponential model. Multiexponential fitting represents this complexity by estimating the lifetimes and fractional contributions of multiple decay components.^355^ However, the estimation becomes ill-conditioned when the number of components increases, the component lifetimes overlap, or the detected photon count is insufficient. Under these conditions, the recovered parameters become strongly dependent on the IRF, initial parameter estimates, and fitting constraints.^356^ Phasor analysis avoids explicit multiexponential fitting and preserves the linear mixing relationship among lifetime components. Nevertheless, a phasor coordinate does not uniquely identify its underlying molecular species. Quantitative molecular assignment therefore requires reference phasors or complementary spectral and biological information. Photon-counting noise also broadens phasor distributions and reduces the separation between fluorophores with closely spaced lifetime signatures.^346^ In macroscopic NIR FLI, these uncertainties are further compounded by photon transport in scattering tissue. Depth-dependent attenuation and temporal dispersion alter the measured temporal profile and bias the recovered lifetime when the analysis does not account for tissue photon transport.^15^ Photon starvation in widefield geometries exacerbates fitting instability and amplifies model-dependent errors, particularly for sub-nanosecond decays.^357^

The limited lifetime range of available NIR fluorophores presents an additional barrier to multiplexing. Several commonly used organic NIR dyes discussed in this review have reported fluorescence lifetimes of ∼0.3 to 2.0 ns under the specific solvent, conjugation, and binding conditions used in the cited studies. This range describes those measurements and does not encompass the full diversity of NIR probe classes. For multiplexing, lifetime separation should therefore be evaluated under matched conditions representative of the intended application.

Clinical translation remains similarly limited, although lifetime contrast could complement a broader range of clinical imaging applications. Current translational efforts are most advanced in FGS, where most clinically deployed systems rely on fluorescence intensity to visualize ICG, 5-ALA-induced PpIX, MB, or targeted NIR probes rather than measuring fluorescence lifetime. Although lifetime measurements have not yet been incorporated into routine clinical FGS, initial clinical studies have demonstrated their potential for tissue discrimination. ICG lifetime has differentiated malignant from noncancerous tissue across several solid tumor types, whereas PpIX lifetime imaging has differentiated brain-tumor and infiltration regions from nonpathological tissue.^30^^,^^31^ However, these studies represent early clinical demonstrations rather than standardized multicenter validation of lifetime-based tissue classification.

Together, inconsistent characterization, acquisition-dependent errors, biological heterogeneity, tissue photon transport, limited lifetime separation, and restricted clinical validation prevent direct comparison of lifetime values across fluorophores, imaging platforms, and biological settings.

## 7 Pathways toward Standardized and Translatable NIR FLI

Addressing the barriers described above requires coordinated progress in measurement standardization, fluorophore characterization, correction of instrument- and tissue-dependent effects, and clinical validation.

A practical first step is the consistent reporting of the experimental and analytical conditions associated with each lifetime measurement. To support reproducibility and cross-study comparison, reports of time-resolved NIR measurements should document the solvent or buffer composition, protein-binding state, temperature, excitation and detection wavelength ranges, detector type, and detected signal quality and background levels. Acquisition-specific information should include excitation repetition rate for pulsed measurements, gate width and temporal sampling for time-gated measurements, and modulation frequency for frequency-domain measurements. When independent repeated measurements are performed, the reported lifetime should be accompanied by the corresponding standard deviation and number of measurements. IRF characterization and alignment, preprocessing procedures, and fitting criteria should also be described when they are included in the analysis.

In addition to consistent reporting, system calibration should include reference fluorophores with independently characterized lifetimes spanning the relevant measurement range. Because reference lifetimes may vary with solvent composition, temperature, concentration, and binding state, calibration measurements should be performed under well-defined conditions and, where possible, using excitation and detection settings comparable to those used for the sample. For imaging systems with appreciable spatial or wavelength-dependent timing variations, channel- or pixel-specific IRF characterization should also be considered.

Parallel to this, a curated open-access database compiling raw decay profiles and metadata under defined experimental conditions would greatly facilitate probe screening, simulation of multiplexed imaging, and training of machine-learning models for lifetime prediction and unmixing. Such a resource is particularly critical for sub-nanosecond NIR fluorophores, where limited photon counts and convolution with the instrument response function make detection and parameter estimation inherently more challenging.

Computational advances provide a broader route toward faster and more robust lifetime recovery. Deep-learning methods have been developed for fit-free lifetime estimation, reconstruction from compressed measurements, and analysis of photon-limited FLI data.^347^^,^^357^^,^^358^ These approaches can reduce reconstruction time and recover lifetime information from measurements for which conventional fitting is limited by sparse photon counts. Future studies should evaluate their generalizability across fluorophores, instruments, IRFs, background levels, and biological specimens. Integrating data-driven reconstruction with physical models of photon statistics, instrument response, and tissue photon propagation may further reduce model-dependent errors and improve the reliability of quantitative NIR FLI.

Future NIR fluorophore development should combine adequate brightness, chemical stability, aqueous compatibility, and biocompatibility with lifetime behavior suited to the intended application. Characterization should address the molecular formulation actually used for imaging, including relevant conjugation and binding states. The required lifetime properties differ among probe identification, environmental sensing, and FRET and should be evaluated against separate performance criteria.

For probe identification and multiplexing, development should prioritize reproducible separation of decay signatures under the anticipated biological and measurement conditions. Expanding the available lifetime range is useful when the resulting signatures remain distinguishable after conjugation, binding, and tissue propagation. Strategies such as site-specific conjugation may help control the local dye environment, but their benefit should be established through measured improvements in reproducibility and unmixing performance. Validation should include mixed samples with unequal signal contributions and report cross-talk and recovery accuracy across relevant photon counts, backgrounds, and tissue depths.

For quantitative environmental sensing, the priority is a selective and reproducible lifetime response over the physiological range of interest. Characterization should establish the calibration relationship, sensitivity, uncertainty, and response time, together with reversibility when repeated measurements of changing conditions are intended. Competing influences, including protein binding, temperature, and aggregation, should be assessed under conditions representative of the application. Where lifetime contrast integrates several environmental influences, it may support biological-state discrimination, but assignment to a specific physicochemical variable requires additional evidence.

For FLI-FRET, probe development should prioritize donor–acceptor pairs with a reproducible donor-only decay and a measurable transfer-induced change under the intended labeling conditions. Validation should establish how labeling density, donor-to-acceptor ratio, and biological environment affect the reference decay and recovered FRET parameters. Appropriate interaction controls are needed to distinguish FRET from unrelated quenching or environmental effects. Reported FRET efficiencies and donor fractions should include uncertainty and demonstrate robustness to the photon counts, background, and instrument response expected in the intended microscopic or macroscopic application.

Translationally, integrating FLI into FGS and diagnostic systems requires coordinated advances in instrumentation, probe characterization, and clinical validation. At the instrumentation level, high-throughput TCSPC, gated optical intensifiers, SPAD arrays, and superconducting nanowire single-photon detectors have expanded acquisition speed, temporal resolution, and detection sensitivity.^358^^–^^362^ Further development should integrate these technologies with standardized calibration and real-time lifetime estimation that remains robust under clinically relevant photon budgets and tissue-scattering conditions. Equally important is the systematic characterization of clinically approved dyes, such as ICG, methylene blue, and Cytalux, under physiological conditions. Establishing these agents as quantitative lifetime biomarkers will require measurements across relevant protein-binding states, concentrations, temperatures, oxygenation levels, and tissue environments. Such measurements would provide condition-specific reference values and define how the physiological environment influences the lifetime of each agent. Clinical validation should incorporate prespecified lifetime-based classification criteria, histopathologic co-validation, assessment of reproducibility across instruments and clinical sites, and prospective evaluation of diagnostic and surgical outcomes. Lifetime-resolved endpoints should also be evaluated separately from intensity-based visualization to determine the independent diagnostic contribution of fluorescence lifetime contrast.

The environmental sensitivity of fluorescence lifetime also provides a basis for extending NIR FLI to vascular and perfusion imaging. Fluorescent vascular probes and endothelial-targeted agents have been used in intensity-based studies of angiogenesis, vascular permeability, edema formation, and tumor-associated vascular remodeling. Protein binding, viscosity, oxygenation, and local biochemical composition alter fluorophore photophysics within vascular environments.^283^^,^^363^^,^^364^ However, the relationship between these factors and lifetime contrast has not been established systematically for NIR vascular imaging. This direction therefore requires controlled studies that isolate individual physiological variables and determine their relationships with the measured lifetime under *in vivo* conditions.

Finally, recent advances extend FLI beyond the NIR spectral window emphasized in this review toward the SWIR region (950 to 2500 nm).^365^^,^^366^ Reduced photon scattering at SWIR wavelengths improves the preservation of spatial information during propagation through tissue.^367^ As organic SWIR fluorophores tend to have even shorter lifetimes compared with organic NIR fluorophores, the long-tail emission of NIR fluorophores provides an alternative approach for lifetime detection in the SWIR range.^87^ Recent studies have demonstrated high-spatiotemporal-resolution FLI in the SWIR window using folate-receptor-targeted ICG nanoprobes, which exhibit prolonged fluorescence lifetimes of ∼750  ps compared with 260 ps for free ICG, enabling quantitative tumor-margin delineation with improved accuracy.^368^ In parallel, *in vivo* SWIR lifetime imaging of whole-body vasculature has been achieved using high-quantum-yield lanthanide-doped nanoparticles, highlighting the value of long-lifetime emitters for deep-tissue imaging and functional vascular mapping.^369^ In addition to modifying existing NIR fluorophores, new SWIR fluorophores such as BT980, BT982, and BT1070 have been developed to reduce quenching, mitigate solvatochromic effects, and improve imaging penetration depth to ∼8  mm.^113^ Although SWIR FLI remains less developed than NIR FLI, these demonstrations establish it as a complementary direction for deep-tissue molecular imaging and image-guided interventions.

## 8 Conclusion

NIR FLI offers a means of investigating molecular interactions and biological environments across microscopic and macroscopic scales. The studies reviewed here highlight that its quantitative value depends on matching probe properties, acquisition methods, and analysis strategies to the intended application. Reported lifetimes must therefore be interpreted in the context of fluorophore formulation, measurement environment, and experimental conditions.

Different applications impose distinct requirements. Probe identification and multiplexing require reproducible decay signatures that remain distinguishable under relevant biological conditions, including unequal signal contributions and tissue-dependent distortions. Environmental sensing requires a calibrated, selective lifetime response over the physiological range of interest, with competing influences characterized. Where several environmental factors contribute, lifetime contrast may support biological-state discrimination without uniquely identifying a specific physicochemical variable. FLI-FRET requires reliable donor-only reference decays and appropriate controls to distinguish energy transfer from other causes of lifetime variation. These differences should guide both probe development and experimental validation.

Acquisition and analysis methods should likewise be selected according to the required output and evaluated through demonstrated precision, bias, photon requirements, acquisition time, and model assumptions. In macroscopic measurements, quantitative interpretation additionally requires assessment of photon-migration effects. Progress toward reproducible NIR FLI will depend on consistent reporting of probe and measurement conditions, accessible reference data, and validation under representative biological conditions. For clinical translation, studies should also establish whether lifetime information improves a defined imaging task. These application-specific criteria provide a practical basis for evaluating new probes and methods and for determining when lifetime contrast supports reliable biological interpretation.

## Data Availability

All data and code supporting the findings of this study are available within the article and its referenced publications.
